# Resveratrol Mediates Anti-Atherogenic Actions In Vitro and in LDL Receptor-Deficient Mice Fed a High-Fat Diet via Antioxidant, Anti-Inflammatory and Plaque-Stabilising Activities

**DOI:** 10.3390/antiox15010076

**Published:** 2026-01-07

**Authors:** Alaa Alahmadi, Reem Alotibi, Yee-Hung Chan, Sarab Taha, Daniah Rifqi, Nouf Alshehri, Sulaiman Alalawi, Fahad Alradi, Alex Gibbs, Timothy R. Hughes, Dipak P. Ramji

**Affiliations:** 1 Cardiff School of Biosciences, Cardiff University, Sir Martin Evans Building, Museum Avenue, Cardiff CF10 3AX, UK; alahmadiag@cardiff.ac.uk (A.A.); alotibirm@cardiff.ac.uk (R.A.); rifqid@cardiff.ac.uk (D.R.); alshehrin@cardiff.ac.uk (N.A.); alalawis@cardiff.ac.uk (S.A.);; 2 Department of Biological Science, College of Science, University of Jeddah, Jeddah 21589, Saudi Arabia; 3 European Cancer Stem Cell Research Institute, Cardiff School of Biosciences, Cardiff University, Hadyn Ellis Building, Maindy Road, Cardiff CF24 4HQ, UK; staha@kfmc.med.sa (S.T.);; 4 Division of Infection and Immunity, Henry Wellcome Building, School of Medicine, Cardiff University, Heath Park, Cardiff CF14 4XN, UK

**Keywords:** atherosclerosis, inflammation, macrophages, resveratrol, gene expression, nutraceuticals, plaque stability

## Abstract

Current pharmacotherapies against atherosclerotic cardiovascular disease are associated with considerable residual risk, together with various adverse side effects. Nutraceuticals, such as resveratrol (RSV), with excellent safety profile, represent promising alternatives and potential treatment. However, the full spectrum of anti-atherogenic actions regulated by RSV and the underlying molecular mechanisms remain poorly understood. The objective of this study therefore was to investigate the impact of RSV on key atherosclerosis-associated processes in monocytes, macrophages, endothelial cells, and smooth muscle cells in vitro, as well as in LDL receptor-deficient mice fed a high-fat diet in vivo. RSV produced beneficial changes in the plasma lipid profile and peripheral blood lymphoid cells in vivo. RSV also attenuated plaque inflammation by decreasing macrophage and T cell content and enhanced markers of plaque stability, with increased levels of smooth muscle cells and collagen content. In vitro, RSV inhibited chemokine-driven monocyte migration, inflammasome activation, matrix metalloproteinase activity, pro-inflammatory gene expression, reactive oxygen species production, and smooth muscle cell invasion. RNA-sequencing of the thoracic aorta revealed key genes and pathways mediating the antioxidant, anti-inflammatory and plaque-stabilising activities of RSV. These studies provide novel mechanistic insights on the anti-atherogenic actions of RSV and support further evaluation in human clinical trials.

## 1. Introduction

Atherosclerotic cardiovascular disease (ACVD) is responsible for most global deaths [1,2]. The disease is associated with a low-grade chronic inflammation of medium and large arteries in response to various atherogenic risk factors, including high plasma levels of low-density lipoprotein (LDL) [1,2]. The pathophysiology of ACVD is associated with several changes in the arterial wall [1,2]: (i) endothelial cells (EC) dysfunction or activation in response to various risk factors leading to chemokine-driven recruitment of immune cells, particularly monocytes that then infiltrate to the subendothelial space where they differentiate into macrophages; (ii) the accumulation of LDL in the subendothelial layer via diffusion and transcytosis and subsequent modification, particularly oxidation, leading to their uptake by macrophages and other cells to form lipid-laden foam cells; (iii) death of foam cells by apoptosis, necrosis, and other processes resulting in the formation of a lipid-rich necrotic core, which triggers stimulation of many pro-inflammatory pathways such as activation of the inflammasome; (iv) migration of smooth muscle cells (SMC) from the media to the intima to form plaque-stabilising fibrous cap containing extracellular matrix (ECM) proteins; (v) increased activity of proteases such as matrix metalloproteinases (MMP) under inflammatory conditions that cause thinning of the fibrous cap; and (vi) plaque rupture leading to subsequent clinical complications such as myocardial infarction and cerebrovascular accidents [1,2].

Current pharmacotherapies against ACVD are not fully effective and associated with considerable residual risk together with various adverse side effects [1,2]. Many emerging therapies also have various limitations such as high costs (e.g., monoclonal antibodies) and side effects (e.g., increased infections for those targeting inflammation) [1,2]. Nutraceuticals, food components with health benefits beyond their nutritional value, generally have excellent safety profile over a prolonged period [1]. However, the mechanisms underlying their protective effects are often poorly understood.

Resveratrol (RSV, 3,5,4ʹ-trihydroxy-*trans*-stilbene) is a natural polyphenol present at high concentrations in the skins of peanuts, red grapes, berries, soybeans, and pomegranates [3,4]. RSV demonstrates an excellent safety profile with doses up to 5 g/day that are well tolerated [4]. Cardio-protective actions of RSV have been demonstrated in vitro, in animal model systems and in humans [3,4,5]. For example, RSV inhibits glucose uptake, modulates glucose, lipid, and amino acid metabolism, reduces lipogenesis, and increases nitric oxide (NO) production in vitro [3,4,5]. In human studies, RSV improves plasma lipid profile and EC dysfunction, reduces inflammatory markers and blood pressure, induces flow-mediated dilation, and inhibits platelet aggregation [3,4,5]. However, clinical trials on RSV in ACVD and other vascular metabolic diseases have produced controversial effects, which in part reflect the very low number of participants, as well as differences in study design, dose, and the duration of intervention [3,4,5]. Whilst many pre-clinical studies on animal models of ACVD have been carried out, they have been rather limited in terms of the parameters analysed and have mainly employed the Apolipoprotein-E (ApoE)–deficient mouse model system [6,7,8,9,10]. However, this is generally regarded as a more aggressive model system for atherosclerosis, as ApoE also has a marked influence on inflammation, haematopoietic stem cell proliferation, monocytosis, and monocyte accumulation in plaques with changes in lipoprotein profile from its deficiency, which are different from those seen in humans [11]. The LDL receptor-deficient (LDLr^−/−^) mouse model overcomes many such limitations and has a more human-like plasma lipid profile that has several characteristics akin to human familial hypercholesterolemia [11]. However, to our knowledge, only a single study on RSV has been carried out in LDLr^−/−^ mice and showed that, whilst *trans*-RSV, the most biologically active and stable isomer, produced alterations in the biomarkers of oxidative stress and lipidaemia, there were no changes in fatty streaks [12]. Such findings highlight the need for more in-depth analyses on the actions of RSV in ACVD in this model system. Therefore, the objective of this study was to investigate the effects of RSV on key atherosclerosis-related processes in monocytes, macrophages, EC, and SMC in vitro, as well as in LDLr^−/−^ mice fed a high-fat diet (HFD) in vivo, and to elucidate the underlying molecular mechanisms.

## 2. Materials and Methods

### 2.1. Materials

Human monocytic THP-1 cell line, human aortic EC (HAEC), and human aortic SMC (HASMC), as well as *trans*-resveratrol (3,4′,5-trihydroxy-*trans*-stilbene, 5-[(1*E*)-2-(4-Hydroxyphenyl)ethenyl]-1,3-benzenediol; ≥99% pure) were from Sigma-Adrich (Gillingham, UK). Lactate dehydrogenase (LDH) assay kit was from Thermo Fisher Scientific (C20301) (Altrincham, UK); Lymphoprep^TM^ was from Stemcell Technologies (Cambridge, UK); interleukin (IL)-1β ELISA kit was from R&D (DLB50) (Abingdon, UK); and monocyte chemotactic protein-1 (MCP-1), interferon-γ (IFN-γ) and tumour necrosis factor-α (TNF-α) were from Peprotech (London, UK). All the other reagents were from Sigma-Aldrich, unless otherwise stated.

### 2.2. Animal Experiments

Male LDLr^−/−^ mice, homozygous for the LDLrtm1Her mutation and backcrossed to the C57BL/6J strain, were originally purchased from the Jackson Laboratory (Bar Harbor, ME, USA) and expanded locally in a pathogen-free and light- and temperature-controlled facility (lights on from 7 a.m. to 7 p.m., 22 °C). Male mice (8-week-old) were randomly assigned to two groups and fed an HFD [21% (*w*/*w*) pork lard and 0.15% (*w*/*w*) cholesterol], alone or mixed with 20 mg/kg/day RSV, for 12 weeks. The concentration of RSV used together with the duration of feeding was based on previous studies [13,14,15,16,17,18]. The concentration of RSV equates to 1.62 mg/kg/day, according to the guide for dose conversion between animals and humans [19,20]. All protocols for the animal studies were carried out following the Guide for Care and Use of Laboratory Animals (NIH Publication No. 85-23; revised 1996) and approved by the Ethics Review Committee of Cardiff University and the United Kingdom Home Office (licence 30/3365 and P5211628).

Mouse body weight was determined at the start of the study and periodically during the feeding period (2 days/week). In addition, the weight of the supplied food and the remaining food was recorded. Peripheral blood (25–50 μL) was collected a day before sacrifice in EDTA microvette tubes for determination of circulating myeloid and lymphoid cells. The mice were sacrificed via CO_2_ asphyxiation, with death confirmed by the absence of a heart pulse. Various adipose tissue depots (brown, subcutaneous, gonadal, inguinal, renal) and organs (heart, spleen, thymus) were weighted, snap-frozen, and stored at −80 °C. Blood from cardiac puncture was collected in tubes containing 50 U/mL heparin and plasma obtained by centrifugation for 10 min at 12,000× *g*. The heart was perfused with PBS, mounted with optimum cutting temperature embedding matrix (Thermo Fisher Scientific, Altrincham, UK), and flash-frozen for subsequent cryosectioning.

### 2.3. Plasma Lipid Profile

The levels of total cholesterol, LDL/very low-density lipoprotein (VLDL)-cholesterol (LDL/VLDL-C), HDL-cholesterol (HDL-C), and cholesteryl esters were determined using the Cholesterol Assay Kit HDL and LDL/VLDL (ab65390), and the levels of triacylglycerol (TG) were determined using the Triglyceride Assay Kit (ab65336), according to the manufacturer’s instructions (Abcam, Cambridge, UK), as described in our previous studies [16,17,18,21].

### 2.4. Analyses of Peripheral Blood Immune Cells

Peripheral blood (12 μL) was transferred to two Eppendorf tubes (for myeloid and lymphoid cell analysis), and red blood cells were lysed by incubation with 600 μL of 1X ammonium chloride (0.8% NH_4_Cl, 0.1 mM EDTA in water buffered with KHCO_3_ to pH of 7.2–7.6) for 8 min. Following centrifugation at 500× *g* for 10 min at 4 °C, the cells were resuspended in 50 μL of antibody mix [phycoerythrin (PE)-conjugated CD115 and PE-cyanine-7 (Cy7)-conjugated Ly6C for monocytes; fluorescein isothiocyanate (FITC)-conjugated Ly6G for granulocytes, PE-conjugated CD3, peridinin-chlorophyll-protein (PerCP)-conjugated CD4, and allophycocyanin (ApC)-Cy7-conjugated CD8 for T cells; PE-Cy7-conjugated NK1.1 for natural killer (NK) cells; and FITC-conjugated B220 for B cells (see Supplementary Table S1 for more details)] and incubated for 30 min at 4 °C. Cells were washed with 1 mL of ice-cold PBS (pH 7.4) supplemented with 2% (*v*/*v*) heat-inactivated foetal calf serum (HI-FCS; 56 °C, 30 min) (referred to as 2% PBS-FCS hereafter), pelleted by centrifugation for 5 min at 500× *g*, resuspended in 300 μL of ice-cold 2% PBS-FCS, transferred to round-bottom polystyrene tubes, and kept on ice. 4′,6-diamidino-2-phenylindole, dilactate (DAPI) nuclear stain was then added immediately before flow cytometric analysis to identify viable cells. In addition, single-stain samples were prepared simultaneously for sample compensation (i.e., adjust cells population to reduce spill-over between channels). Samples were analysed until 20,000 counts were reached. Flow cytometry was carried out on a BD LSRFortessa^TM^ Lasers flow cytometer (BD Biosciences, Wokingham, UK) with data analyses using the FlowJo v.10 software (see Supplementary Figures S1 and S2 for the gating strategy).

### 2.5. Plaque Analyses

Sequential sectioning of the aortic root (8 μm), together with analyses of plaque size and lipid content using Oil Red O, collagen content using Van Geison’s stain, and the content of macrophages, CD3 T cells, and SMC via immunofluorescent staining, was carried out as our previous studies [16,17,18]. All image analyses were carried out in a blinded fashion using ImageJ software, version 2.0.0-rc-69/1.52p with various plaque parameters determined as our previous studies [16,17,18].

### 2.6. RNA-Sequencing (RNA-Seq)

Total RNA was purified from the thoracic aorta, which had been previously stored in RNAlater^TM^ stabilisation solution (Thermo Fisher Scientific, Altrincham, UK) at −80 °C, using TissueLyser II with one 2 mm stainless steel beads and the RNeasy Mini Kit, according to the manufacturer’s instructions (Qiagen, Manchester, UK). The RNA (RNA integrity number > 6.8) was sent to Novogene (Cambridge, UK) for RNA-seq with standard bioinformatic analyses. This included additional RNA quality check, mRNA library preparation (poly A enrichment), and Illumina sequencing PE150, followed by data quality control and data filtering, and standard bioinformatic analyses, including mapping to reference genome, gene expression quantification and correlation analysis, differential expression analysis, and enrichment analysis [e.g., Gene Ontology (GO) and Kyoto Encyclopaedia of Genes and Genomes (KEGG) pathways] of differentially expressed genes (DEGs). In addition, the Ingenuity Pathway Analysis (IPA) software (Qiagen, Manchester, UK) (https://www.qiagen.com/ja-us/products/discovery-and-translational-research/next-generation-sequencing/informatics-and-data/interpretation-content-databases/ingenuity-pathway-analysis, accessed on 12 August 2022) was used for a more in-depth analysis of DEGs [*p*-adjusted (*p*adj.) < 0.05 filter], such as affected canonical pathways, diseases and function, and upstream regulators. Heatmaps were generated using the Heatmapper online software (http://www.heatmapper.ca/expression/, accessed on 12 August 2022).

### 2.7. Cell Culture

HASMC and HAEC were cultured in their respective ready-to-use media, according to the manufacturer’s instructions (Sigma-Adrich, Gillingham, UK). Primary cultures of human monocyte-derived macrophages (HMDMs) were obtained from monocytes of buffy coats, as our previous studies [16,17,22]. Culturing of HMDM and THP-1 cells, together with differentiation of the latter using 0.16 μM phorbol 12-myristate 13-acetate for 24 h, was performed, as our previous studies [16,17,22].

### 2.8. In Vitro Assays

Assessment of cell viability using the LDH Cytotoxicity Assay Kit (Thermo Fisher Scientific, Altricham, UK), MCP-1-driven monocytic migration using modified Boyden chambers (Thermo Fisher Scientific, Altricham, UK), platelet-derived growth factor (PDGF)-induced invasion of HASMCs, reactive oxygen species (ROS) production using the 2′,7′–dichorofluorescin diacetate (DCFDA) cellular ROS detection kit (Abcam, Cambridge, UK; ab113851), MMP activity using fluorescence resonance energy transfer-based assay (Abcam, Cambridge, UK; ab112147), and cholesterol-crystal-mediated production of IL-1β using an ELISA kit (R&D, Abingdon, UK) were carried out, as our previous studies [16,17,18,21,22]. Initial studies used several doses of RSV (0 μM, 25 μM, 50 μM, 75 μM, and 100 μM), and, from the outcomes, subsequent studies used concentrations of 25 μM and/or 50 μM. Total RNA was prepared using RiboZol^TM^ (Avantor, Lutterworth, UK); real-time quantitative PCR was performed using primers for MCP-1 (5′-CGCTCAGCCAGATGCAATCAATG-3′ and 5′-TGGTCTTGAAGATCACAGCTTCTTTGG-3′) and glyceraldehyde 3-phosphate dehydrogenase (GAPDH) gene (5′-CTTTTGCGTCGCCAGCCGAG-3′ and 5′-GCCCAATACGACCAAATCCGTTGACT-3′), and data analysis via the ΔΔct method was performed, as our previous studies [16,17,18,22]. Mitochondrial superoxide (MitoSox) production was determined using the MitoSOX^TM^ Red mitochondrial superoxide indicator according to the manufacturer’s instructions (Thermo Fisher Scientific, Altrincham, UK; MC36008).

### 2.9. Statistical Analyses of Data

Normality of the data was determined using the Shapiro–Wilk test. Statistical analysis of more than two groups with normally distributed data was carried out by one-way analysis of variance (ANOVA), followed by either Tukey’s (for equal variances) or Dunnett’s (for unequal variances) post hoc test. For data with two groups only, an unpaired *t*-test (for normally distributed data) or Mann–Whitney U test (for not normally distributed data) was used. Analysis was carried out on GraphPad Prism 9 software, where significance was defined by *p* ≤ 0.05.

## 3. Results

### 3.1. RSV Decreases Plaque Inflammation and Produces a Stable Plaque Phenotype in LDLr^−/−^ Mice Fed an HFD

**Figure 1:**
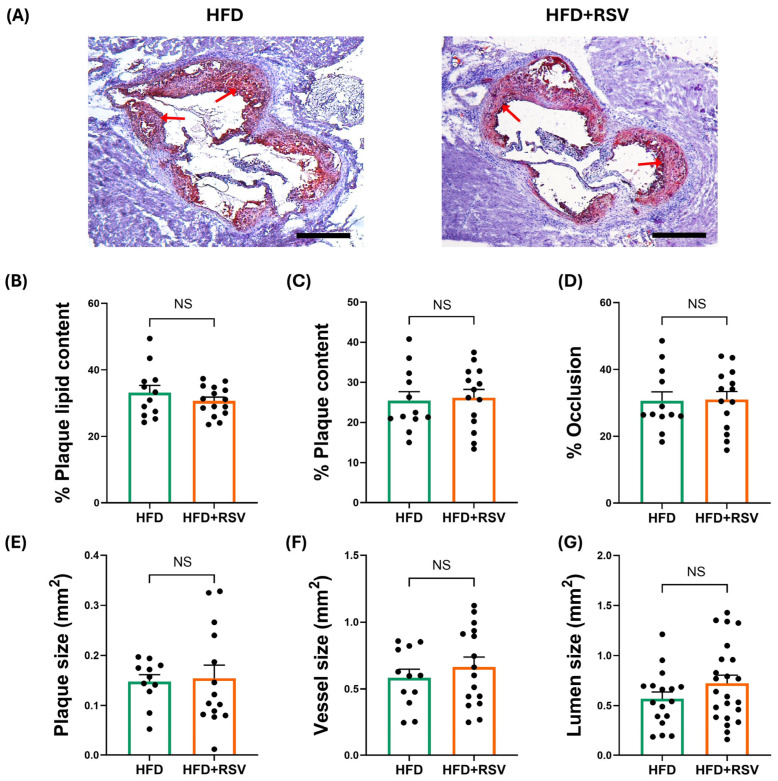
RSV has no effect on plaque burden and lipid content in LDLr^−/−^ mice fed an HFD. The mice were fed either HFD (control) or HFD supplemented with RSV for 12 weeks, and aortic root sections were stained with Oil Red O (ORO). Images were captured using a Leica DMRB microscope (Leica Microsystems, Milton Keynes, UK) under ×5 magnification and analysed using ImageJ software (arrows indicate ORO staining in plaques). Representative images are shown in panel (**A**) (scale bar of 400 μm), with graphs indicating lipid content (determined as percentage ORO^+^ staining; (**B**)); plaque content (calculated as percentage plaque area of vessel area; (**C**)); occlusion (calculated as percentage plaque area of lumen area; (**D**)); plaque size (**E**); vessel size (**F**); and lumen size (**G**). Data are presented as mean ± SEM from n = 12 (**B**–**D**,**F**), 11 (**E**), or 17 (**G**) for HFD group and n = 15 (**B**), 14 (**C**–**E**), 16 (**F**), or 23 (**G**) for HFD + RSV group. Statistical analysis was performed using an unpaired Student’s *t*-test. NS—not significant.

**Figure 2:**
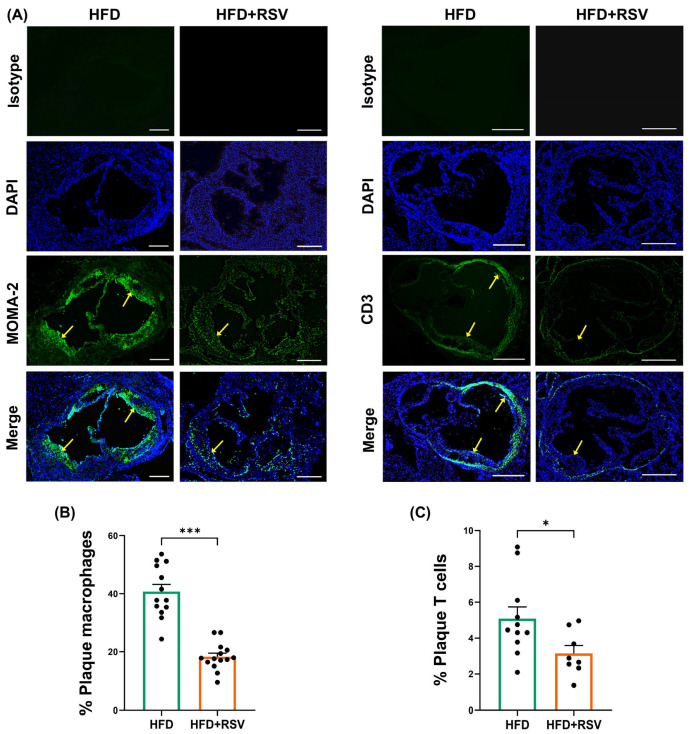
RSV attenuates plaque content of macrophages and CD3+ T cells. Immunofluorescence staining was carried out on sections from the aortic root of LDLr^−/−^ mice fed either HFD or HFD supplemented with RSV. Images were captured using an Olympus BX61 microscope (Evident Scientific, Stansted, UK) under ×4 magnification and analysed using ImageJ software (arrows indicate macrophage/CD3-T cell staining in plaques). Representative images are indicated in panel (**A**) (scale bar of 400 μm), with graphs showing plaque content of MOMA-2+ macrophages (n = 13 for HFD group and n = 14 for HFD + RSV group; (**B**)) and CD3+ T cells (n = 11 for HFD group and n = 8 for HFD + RSV group; (**C**)). Data are presented as mean ± SEM, with statistical analysis performed using an unpaired Student’s *t*-test, where * *p* ≤ 0.05 and *** *p ≤* 0.001.

**Figure 3:**
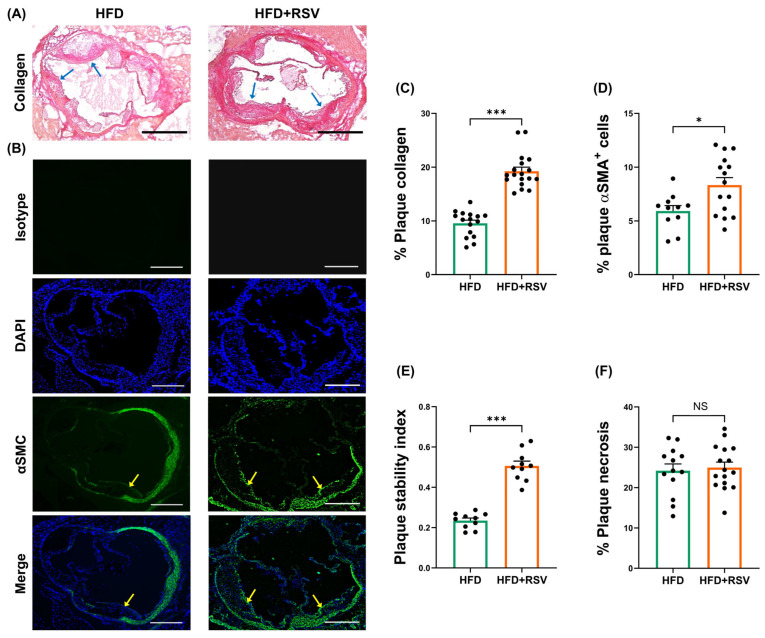
RSV increases markers of plaque stability. LDLr^−/−^ mice were fed either HFD or HFD supplemented with RSV for 12 weeks, and sections from the aortic root were subjected to Van Gieson’s staining to determine the collagen content within the plaque. Images were captured using Leica DMRB microscope (Leica Microsystems, Milton Keynes, UK) under ×5 magnification (representative images with scale bar of 400 μm is shown in panel (**A**); arrows indicate collagen staining in plaques). In addition, immunofluorescence staining was carried out to detect *α*-smooth muscle actin (*α*SMA)^+^ in plaques (representative images with scale bar of 400 μm are shown in panel (**B**); arrows indicate *α*SMA^+^ staining in plaques). Graphs (mean ± SEM) show plaque content of collagen (n = 16 for HFD group and n = 18 for HFD + RSV group; (**C**)), *α*SMA^+^ cells (n = 11 for HFD group and n = 15 for HFD + RSV group; (**D**)), plaque stability index calculated as (smooth muscle cells + collagen)/(macrophages + lipids) (n = 10 for HFD group and n = 10 for HFD + RSV group; (**E**)), and necrosis calculated as acellular regions within the plaque (n = 13 for HFD group and n = 16 for HFD + RSV group; (**F**)). The images were analysed using ImageJ software, and statistical analysis was performed using an unpaired Student’s *t*-test, where NS—not significant, * *p* ≤ 0.05 and *** *p* ≤ 0.001.

The effects of RSV on the HFD-induced progression of atherosclerosis were first determined. RSV had no significant effect on plaque lipid content, plaque content (percentage plaque area of vessel area), occlusion (percentage plaque area of lumen area), plaque size, vessel size, and lumen size (Figure 1). In contrast, there was a significant reduction in the plaque content of macrophages (*p* ≤ 0.001) and CD3+ T cells (*p* = 0.036) (Figure 2). In addition, RSV produced a significant increase in the plaque content of collagen (*p* ≤ 0.001) and SMC (*p* = 0.015) (Figure 3A–D). These changes were associated with a significant increase in the plaque stability index (*p* ≤ 0.001), without any change in plaque necrosis (Figure 3E,F). Overall, these results demonstrate that RSV attenuates plaque inflammation and produces a stable plaque phenotype. The potential molecular mechanisms underlying such beneficial changes were investigated further in vivo and in vitro.

### 3.2. RSV Improves Plasma Lipid Profile and Immune Cell Profile in the Peripheral Blood of LDLr^−/−^ Mice Fed an HFD

RSV had no significant effect on the final mouse weight, HFD-induced weight gain, or weight of total adipose tissue, including total white adipose tissue and total brown adipose tissue, as well as subcutaneous, gonadal, inguinal, or renal adipose tissue depots (Table 1). In addition, RSV had no significant effects on the weight of spleen or thymus (Table 1). In contrast, there was a trend towards reduction in the weight of the heart (*p* = 0.054), though there was no change in the cardiac hypertrophy index, as determined by dividing the heart weight (mg) with the tibia length (mm) (Table 1).

For the plasma lipid profile, RSV had no effect on the levels of TG, HDL-cholesterol (HDL-C), and free cholesterol (FC) but produced significant reductions in the levels of LDL/VLDL-cholesterol (LDL/VLDL-C), major pro-atherogenic lipoproteins [2], and cholesteryl esters (CE) (*p* ≤ 0.001 in both cases), with a trend towards reduction in the levels of total cholesterol (TC) (*p* = 0.069) (Table 1).

For the peripheral blood immune cell profile, RSV produced a trend towards reduction in the levels of B cells (*p* = 0.068), T cells (*p* = 0.082), and CD8+ T cells (*p* = 0.089) without affecting the levels of CD4+ T cells, NK cells, monocytes, Ly6C^High^ monocytes, Ly6C^low^ monocytes, or granulocytes (Table 1). Thus, the anti-inflammatory actions of RSV seen in plaques extended to the peripheral blood.

### 3.3. RSV Has Many Anti-Atherogenic Actions on Monocytes/Macrophages, EC and SMC In Vitro

**Figure 4:**
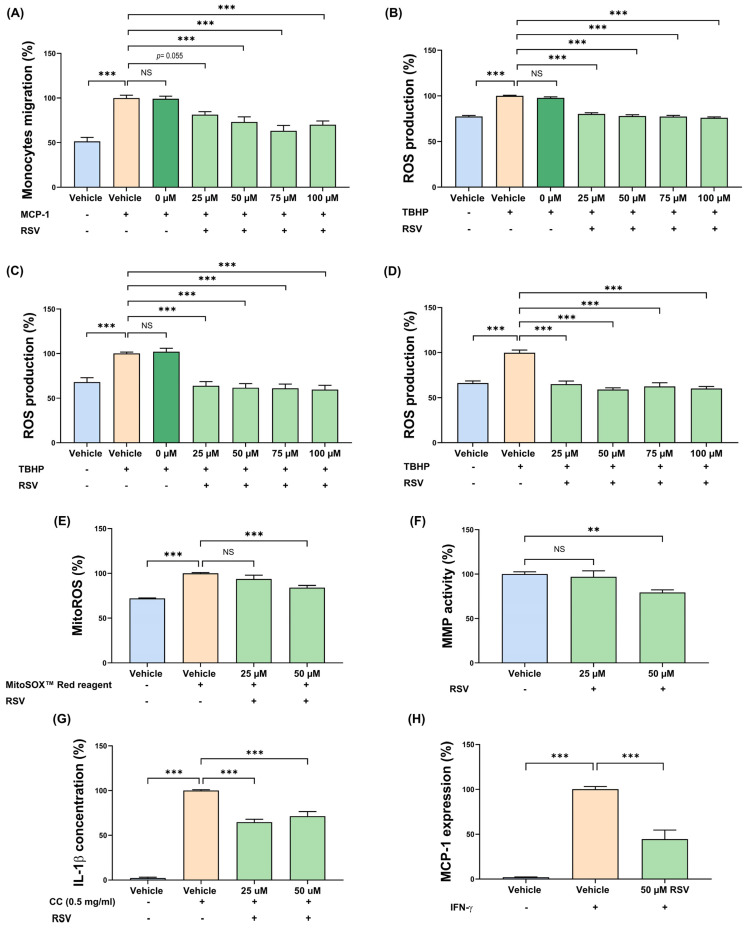
RSV has several anti-atherogenic actions on key monocyte/macrophage processes implicated in atherosclerosis. For migration, THP-1 monocytes were incubated with vehicle or MCP-1 (20 ng/mL), with the indicated concentration of RSV for 3 h. The proportion of migrated cells were expressed as a percentage of total input cells and displayed as a percentage of migration relative to the vehicle control, which was arbitrarily set to 100% (**A**). For ROS production, THP-1 monocytes (**B**), THP-1 macrophages (**C**), and HMDMs (**D**) were treated with TBHP and then either vehicle (control) or RSV for 3 h. Cells treated with vehicle in the absence of TBHP were also included for comparative purposes. ROS production is displayed as a percentage to the vehicle control, which was arbitrarily set to 100%. The effect of RSV on mitoROS production in THP-1 macrophages was investigated at 24 h using MitoSOX^TM^ Red staining in which mitochondrial superoxide production was measured (values from vehicle-treated cells arbitrarily assigned as 100% (**E**)). For MMP activity, THP-1 macrophages were treated with either the vehicle or RSV for 3 h, and the protease activity was determined as a percentage to the vehicle control which was set to 100% (**F**). Cholesterol crystal-induced inflammasome activation was assessed in THP-1 macrophages at 24 h by monitoring the IL-1β secretion by ELISA and expressed as a percentage to the vehicle control that was arbitrarily set to 100% (**G**). The expression of *MCP-1* was assessed in IFN-γ-stimulated THP-1 macrophages that were either treated with vehicle (vehicle control) or 50 μM RSV for 24 h (**H**). For comparative purposes, unstimulated cells with vehicle only were included. Gene expression levels were determined by qPCR using a comparative ΔΔCT method and normalised to the housekeeping gene (*GAPDH*), with values from cells treated with vehicle and IFN-γ arbitrary assigned as 100%. Data are presented as mean ± SEM from three (**E**,**G**), four (**A**–**D**), or five (**F**,**H**) independent experiments. Statistical analysis was performed using a one-way ANOVA with Tukey’s post hoc analysis (**A**,**B**,**D**) or Dunnett post hoc test (**C**,**E**–**H**), where NS—not significant, ** *p ≤* 0.01 and *** *p* ≤ 0.001.

Experiments on cell culture model systems in vitro were carried out to further probe the molecular mechanisms underlying the anti-inflammatory and plaque-stabilising phenotype seen in vivo. Monocyte-derived macrophages play pivotal roles at all stages of atherosclerosis [2]. The effect of RSV on relevant atherosclerosis-associated monocyte/macrophage processes were first investigated using the THP-1 cell line, which is widely used for the study of human monocytes and macrophages in the disease with demonstrated conservation of responses with primary cultures and in vivo [16,17,22,23], with key findings confirmed in primary cultures of HMDM. The studies focussed on inflammation and ROS production, where our initial studies used RSV at several concentrations (0 μM, 25 μM, 50 μM, 75 μM, and 100 μM), which had no effects on the viability of both THP-1 macrophages and HMDM (Supplementary Figure S3A,B). Migration of monocytes in response to key chemokines such as MCP-1 is a critical early event in the pathogenesis of atherosclerosis that leads to their recruitment and subsequent accumulation of macrophages in plaques and associated inflammatory response [16,17,18,22,24]. RSV significantly attenuated the MCP-1-driven monocytic migration at 50 μM, 75 μM, and 100 μM (*p* ≤ 0.001 in all cases) and produced a trend towards reduction at 25 μM (*p* = 0.055; Figure 4A). The ROS-mediated oxidation of LDL and subsequent inflammation is another critical event in the pathogenesis of atherosclerosis [24], and hence the effects of RSV on tert-butyl hydroperoxide (TBHP)-induced ROS production (i.e., mimicking ROS generation in pathological conditions) in human THP-1 monocytes and macrophages were determined. RSV attenuated the TBHP-induced ROS production in both THP-1 monocytes and macrophages at 25 μM, 50 μM, 75 μM, and 100 μM (*p* ≤ 0.001 in all cases) (Figure 4B,C). To rule out the possibility that these results were because of the use of the THP-1 cell line, the experiments were repeated in primary HMDM. RSV inhibited the TBHP-mediated ROS production in HMDM at all concentrations used (*p* ≤ 0.001 in all cases; Figure 4D).

As the two lowest concentrations of RSV (25 μM and 50 μM) inhibited the MCP-1-driven monocytic migration and the TBHP-induced ROS production, they were used for subsequent studies on macrophages. Mitochondria are increasingly being identified to play an important role in atherosclerosis, in part via ROS production [25]. The effect of RSV on MitoSox production in THP-1 macrophages was hence determined and found to be inhibited at the 50 μM concentration (*p* ≤ 0.001; Figure 4E). MMPs produced by macrophages play a critical role in the degradation of ECM, and thereby plaque stability [26]; hence, the effect of RSV on MMP activity was determined at both 3 and 24 h to delineate both short-term and longer-term actions/effects. A significant inhibition of MMP activity was only seen at the 50 μM concentration at 3 h (Figure 4F; *p* = 0.004). Cholesterol crystals activate the nucleotide-binding domain, leucine-rich–containing family, pyrin domain–containing-3 inflammasome, leading to the secretion of IL-1β that contributes to the chronic inflammation seen in atherosclerotic plaques [27]. The effect of RSV on such IL-1β production was hence determined in THP-1 macrophages. As expected, cholesterol crystals increased IL-1β secretion, and this was inhibited by 25 μM and 50 μM RSV (*p* ≤ 0.001 in both cases; Figure 4G). Because mitochondrial superoxide production and MMP activity were only inhibited by 50 μM ROS, this concentration was used in subsequent studies. IFN-γ is a key pro-atherogenic cytokine that induces the expression of *MCP-1*, which contributes to the chronic inflammatory response by the recruitment of immune cells [24], and this was inhibited by 50 μM RSV in THP-1 macrophages (*p* ≤ 0.001; Figure 4H).

**Figure 5:**
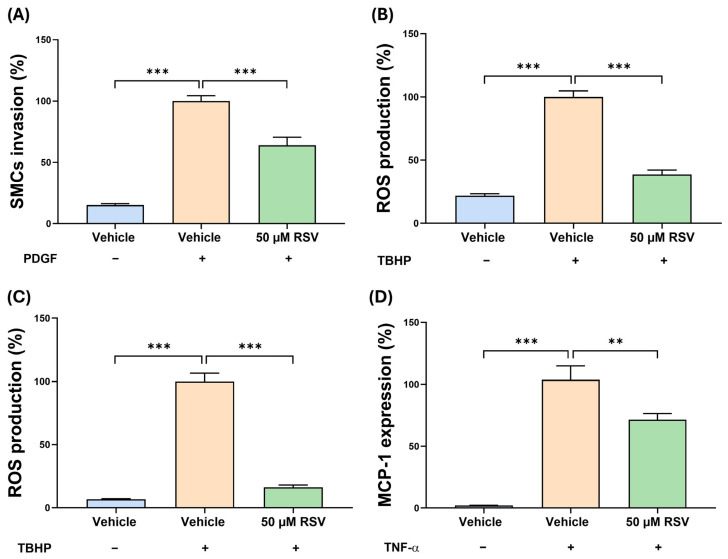
RSV has beneficial effects on HASMCs and HAECs. The effect of RSV on the invasion of SMC was assessed using HASMCs incubated with PDGF-BB and either the vehicle control (vehicle) or RSV for 4 h. Cells incubated with the vehicle in the absence of PDGF-BB (No PDGF) were included for comparative purposes. The number of migrated cells were counted and averaged per five high-power field and presented as a percentage to the vehicle control, which was arbitrarily set to 100% (**A**). For ROS production, HASMCs (**B**) and HAECs (**C**) were treated with TBHP and either the vehicle (control) or 50 µM RSV for 3 h. Cells treated with vehicle in the absence of TBHP were included for comparative purposes. ROS production is displayed as a percentage to the vehicle control, which was arbitrarily set to 100% (**B**,**C**). For pro-inflammatory gene expression in HAECs, the expression of *MCP-1* was assessed following TNF-α stimulation for 24 h with vehicle (control) or 50 μM RSV for 24 h. Cells incubated with vehicle alone were also included for comparison. Gene expression levels were determined by qPCR using a comparative ΔΔCT method and normalised to the housekeeping gene (*GAPDH*), with values from cells treated with vehicle and TNF-α arbitrary assigned as 100% (**D**). Data are presented as mean ± SEM from three independent experiments, and statistical analysis was performed using a one-way ANOVA with Dunnett post hoc test, where ** *p ≤* 0.01 and *** *p ≤* 0.001.

SMC and EC also contribute to plaque inflammation and stability [2]; hence, the effect of RSV on HASMCs and HAECs was investigated using 50 μM RSV, which had no effect on the viability of these cells (Supplementary Figure S4). PDGF produces pathological migration of SMCs, and this was significantly inhibited by RSV (*p* ≤ 0.001; Figure 5A). As mentioned above, ROS production is a key mediator of inflammation, and RSV significantly inhibited the TBHP-induced ROS production in HASMCs (*p* ≤ 0.001; Figure 5B). Similar inhibition of TBHP-induced ROS production was also seen in HAECs (*p* ≤ 0.001; Figure 5C). TNFα is a key cytokine that causes EC dysfunction and subsequent inflammation via increased expression of the *MCP-1* gene [24]. RSV significantly inhibited the TNF-α-induced pro-inflammatory *MCP-1* expression in HAECs (*p* = 0.007; Figure 5D).

### 3.4. RNA-Seq Analyses of the Thoracic Aorta Identifies Key Genes and Pathways That Are Potentially Involved in the Beneficial Actions of RSV on the Progression of Atherosclerotic Plaques

**Figure 6:**
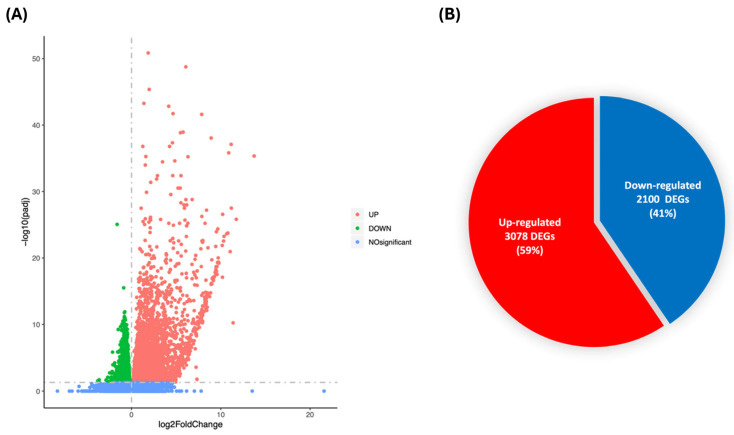
Volcano plot and Venn diagram of differentially expressed genes. (**A**) Volcano plot showing upregulated and downregulated DEGs in which the x-axis represents the log2Fold change, while the y-axis represents −log 10 adjusted *p*-value (<0.05 was used as cut-off). The blue dots represent non-significant DEGs, red dots represent significantly upregulated DEGs, while green dots represent significantly downregulated DEGs. (**B**) The Venn diagram represents the number and percentage of significantly upregulated DEGs (red) and downregulated DEGs (blue) in RSV group after 12 weeks of feeding HFD compared to the HFD only control group (n = 5 for HFD group and n = 4 for HFD + RSV group).

To further delineate the mechanisms underlying the anti-inflammatory and plaque-stabilising effects of RSV seen in vitro and in vivo, RNA-seq of the thoracic aorta was carried out to identify key genes and pathways that are potentially involved in the protective actions of this polyphenol. Overall, there were 5178 DEGs significantly different between the two groups (*p*adj. < 0.05), with 3078 upregulated and 2100 downregulated (Figure 6A,B). A list of the top 20 upregulated and downregulated genes, together with their proposed function from IPA, is shown in Table 2 and Table 3 and includes those involved in the regulation of immune and inflammatory responses [e.g., *Scgb1a1* (*Secretoglobin*, *family 1A*, *member 1*; *p*adj. 1.52 × 10^−26^), *Bpifa1* (*BPI fold containing family A*, *member 1*; *p*adj. 5.76 × 10^−11^), *Bpifb1* (*BPI fold containing family B member 1*; *p*adj. 8.04 × 10^−38^), *Sftpd* (*Pulmonary surfactant-associated protein D*; *p*adj. 2.81 × 10^−23^), *Scgb3a1* (*Secretoglobin*, *family 1A*, *member*; *p*adj. 9.60 × 10^−21^), *Ctse* (*Cathepsin E*; *p*adj. 0.043), *Themis* (*Thymocyte-expressed molecule involved in selection-1*; *p*adj. 0.045)], cell migration and adhesion [e.g., *Esrp1* (*Epithelial splicing regulatory protein 1*; *p*adj. 3.01 × 10^−18^), *Adam4* (*a disintegrin and metallopeptidase domain 4*; *p*adj. 0.020)], metabolism [e.g., *Ppp1r3g* (*Protein phosphatase 1 regulatory subunit 3G*; *p*adj. 0.033)], and EC transcytosis [e.g., *Mfsd2a* (Lysolipid transporter A, lysophospholipid; *p*adj. 0.002)].

**Figure 7:**
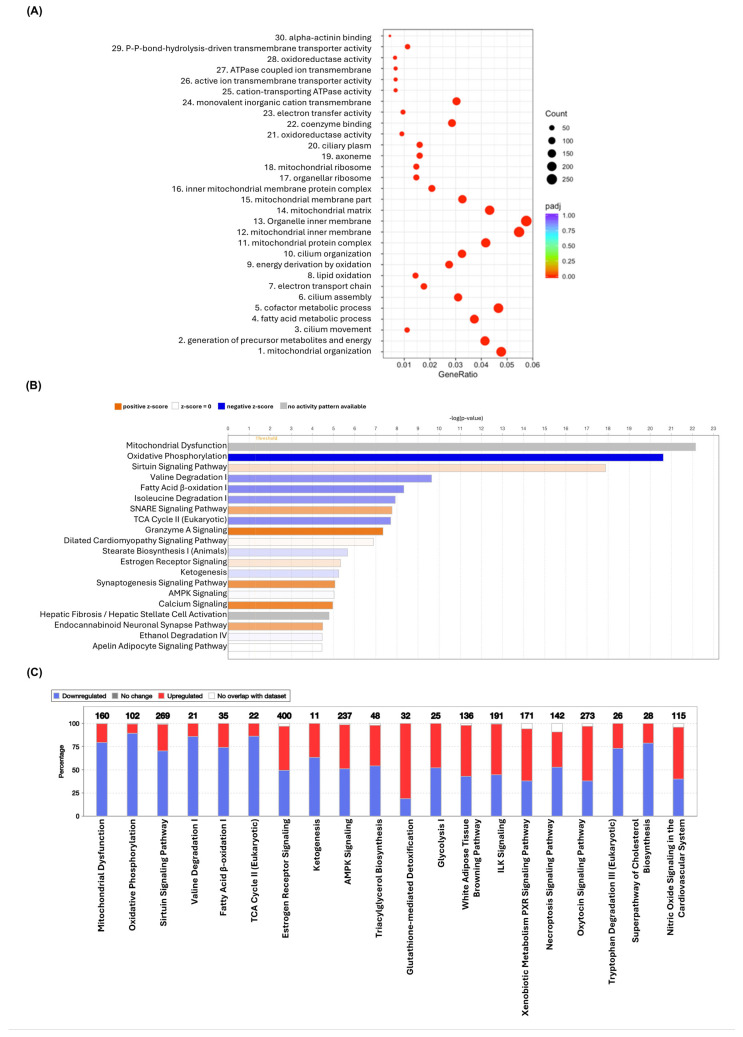
Effects of resveratrol supplementation on canonical pathways and GO analysis. (**A**) GO enrichment analysis dot plot showing the affected terms by RSV intervention. The abscissa in the graph is the ratio of the differential gene number to the total number of differential genes on the GO term, and the ordinate is the top 30 significant GO enrichment terms. The colour of the dots represents the significant level of enrichment, while the size of the dots represents the number of genes annotated to a specific GO term. (**B**) The top 20 significantly affected canonical pathways by RSV intervention. The names of the pathways are displayed on the y-axis, while the x-axis shows the −log of *p*-value that is calculated by the right-tailed Fisher’s Exact Test. The taller the bars, the more significant the pathway. Z-score calculation uses the match between observed gene expression changes and expected patterns from the literature curated in the IPA knowledge base. The blue and orange bars represent an overall negative z-score (predicted downregulated) and the positive z-score (predicted upregulated), respectively. The intensity of the blue or orange bars corresponds to the strength of the prediction often related to the absolute z-score (i.e., the darker/more intense colour indicates a stronger prediction for a higher absolute positive or negative z-score). The grey bar indicates pathway that IPA is unable to make a prediction, while the white bar shows the pathway with a z-score of zero or close to zero. The thin orange vertical line represents the ratio that is calculated as number of genes in a given pathway that meet cut-off criteria/the total number of known genes that make up that pathway and found in IPA reference gene set. (**C**) The stacked bar chart shows canonical pathways specifically implicated in atherosclerosis with up- and downregulated genes (red and blue, respectively). The white area of the bar represents genes involved in the pathway but are not in the dataset. The numerical value above each bar represents the total number of genes in the pathway. Image produced using the IPA programme (n = 5 for HFD group and n = 4 for HFD + RSV group).

GO enrichment analysis allows annotation of DEGs according to their biological processes, cellular components, and molecular functions. The 30 most significant terms with indications of number of genes in each GO term are shown in Figure 7A and include those implicated in metabolism, mitochondrial organisation, and lipid oxidation. IPA software was used to further probe canonical pathways for transcripts that are differentially activated or inhibited in the RSV group compared to the control group with adjusted *p* < 0.05. The top 20 canonical pathways affected by RSV are shown in Figure 7B, whereas the top 20 potential pathways specifically implicated in the pathogenesis of atherosclerosis with numbers of genes in each pathway are shown in Figure 7C. Overall, RSV downregulates several pathways such as oxidative phosphorylation, valine and tryptophan degradation, fatty acid β-oxidation, tricarboxylic acid (TCA) cycle, TG and cholesterol biosynthesis, ketogenesis, glycolysis, and necroptosis signalling pathways. On the other hand, RSV upregulates sirtuin signalling pathway, antioxidant glutathione-mediated detoxification, white adipose tissue browning pathway, integrin-linked kinase signalling, and NO signalling.

Although mitochondrial dysfunction ranked at the top of the canonical pathways list (*p* = 7.24 × 10^−23^) and was significantly enriched in our dataset, IPA lacked sufficient directional information to predict whether this pathway was activated or inhibited. Oxidative phosphorylation was the second significantly enriched canonical pathway inhibited by RSV (*p* = 2.43 × 10^−21^). All the proteins encoded by the corresponding genes are located in the inner mitochondrial membrane and function as enzymes or transporters (Supplementary Figure S5). The sirtuin signalling pathway emerged as the third significantly enriched canonical pathway and the first significantly activated pathway by RSV (*p* = 1.32 × 10^−18^).

As RSV had major anti-inflammatory actions in vitro and in vivo, IPA was employed to investigate the effects of RSV on key inflammatory signalling pathways implicated in atherosclerosis with the significant DEGs overlayed on the pathways to visualise how the significantly up- and downregulated genes in the dataset impacted pathway activation. RSV was predicted to significantly inhibit the pro-inflammatory nuclear factor kappa-light-chain-enhancer of activated B cells (NF-κB) signalling pathway (Supplementary Figure S6), T cell signalling pathway (Supplementary Figure S7), which is consistent with decreased T cell content in atherosclerotic plaques (Figure 2), and the NLRP3 inflammasome pathway (Supplementary Figure S8), which is consistent with reduced activation seen in macrophages in vitro (Figure 4G). On the other hand, the canonical Wingless and int-1 (Wnt)/β-catenin pathway was predicted to be activated (Supplementary Figure S9).

**Figure 8:**
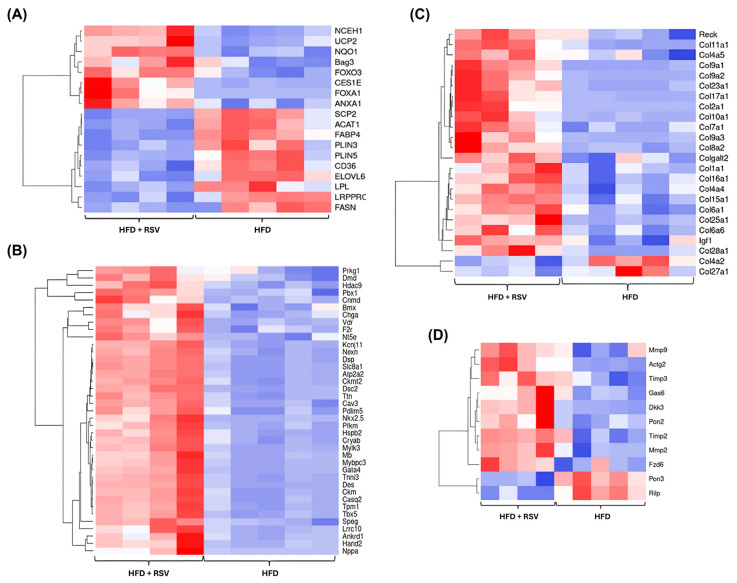
Heatmaps for key genes implicated in specific biological functions impacted by RSV intervention. The list of genes whose expression was significantly regulated and associated with a biological process or function from IPA was pre-ranked from lowest to highest log2FC and then inputted into Heatmapper software as described in Section 2.6. The colour gradient represents gene expression levels with red indicating higher levels and blue showing lower levels. (**A**) genes implicated in the regulation of lipid metabolism, (**B**) genes implicated in the improvement of cardiac function, (**C**) genes associated with collagen synthesis, and (**D**) genes involved in the regulation of atherosclerotic plaque stability (n = 5 for HFD group and n = 4 for HFD + RSV group).

The Tox function tool in the IPA, together with heat map analyses of associated DEGs, were used to further probe the RNA-seq data. Consistent with a beneficial plasma lipid profile, together with an anti-atherogenic and plaque-stabilising phenotype, RSV supplementation was found to impact key genes implicated in lipid metabolism (Figure 8A), improvement of cardiac function (Figure 8B), collagen synthesis (Figure 8C), and those associated with plaque stability (Figure 8D).

Finally, upstream regulators that are potentially involved in the observed changes in gene expression were identified, together with whether they are likely to be activated or inhibited (Table 4). Thioredoxin reductase-1 (Txnrd1) is an example of an upstream regulator that was predicted to be inhibited by RSV intervention. Network analysis demonstrated its ability to regulate key genes implicated in antioxidant activities, with downstream inhibition of inflammation and lipid metabolism (Supplementary Figure S10).

## 4. Discussion

Despite many studies indicating cardio-protective actions of RSV [4,5,6,7,8,9,10], in-depth understanding of the underlying mechanisms remains relatively poor. We show here that RSV produces a beneficial plasma lipid profile, attenuates plaque inflammation, and produces a stable plaque phenotype (Table 1 and Figure 2 and Figure 3). The anti-inflammatory action of RSV extends to the peripheral blood in vivo (Table 1). In vitro studies provide mechanistic insights for the protective actions of RSV, which include the inhibition of chemokine-driven monocytic migration, pro-inflammatory gene expression, ROS production, inflammasome activation, MMP activity, and PDGF-induced migration of SMCs (Figure 4 and Figure 5). In addition, RNA-seq of the thoracic aorta identifies key genes and pathways for the protective actions of RSV that included beneficial effects against oxidative stress, inflammation, metabolism, and plaque stability (Figure 6, Figure 7 and Figure 8). Taken together, these studies provide novel insights into the athero-protective actions of RSV, together with the potential underlying mechanisms.

Previous studies on RSV in relation to atherosclerosis have been restricted mainly to the ApoE^−/−^ model system [5,6,7,8,9,10], which has several limitations [11], where changes in plaque burden and/or lipid content were observed. However, only a single study has been carried out on the LDLr^−/−^ model system [12], and consistent with our studies, no effects were seen at the level of plaque lipid content. However, this study did not analyse plaque cellular and collagen content, which we found to be favourably impacted by RSV (i.e., reduced macrophage and T cell content and increased SMC and collagen content) (Figure 2 and Figure 3). The reduction in T cells in the plaque is likely to be attributable to a decrease found within the peripheral blood (Table 1). T cells release the cytokine IFN-γ, which has been shown to enhance plaque progression and reduce stability [24]. Thus, inhibition of the actions of IFN-γ and other pro-inflammatory cytokines may be a key contributor to the anti-atherogenic actions of RSV. Indeed, RSV inhibited the IFN-γ- and TNF-α-induced MCP-1 expression (Figure 4H and Figure 5D); the latter cytokine is involved in EC dysfunction [24], together with the MCP-1-stimulated monocytic migration (Figure 4A), which has a profound impact on plaque macrophage accumulation. IFN-γ also decreases the differentiation of monocytes to macrophages [24]; therefore, a decrease in this cytokine via T cells may also have impacted plaque macrophage content, especially since no changes in the levels of Ly6C^high^ monocyte levels were seen in the peripheral blood (Table 1). RSV produced a marked increase in plaque stability index, with no effect on plaque necrosis (Figure 3E,F), and this was associated with a reduction in MMP activity (Figure 4F), modulation in the expression of collagen and other plaque-stabilising genes (Figure 8C,D), and a reduction in potentially pathological invasion of SMC produced by PDGF (Figure 5A). Indeed, inhibition of the expression of PDGF or its receptor or subsequent signal transduction pathways has been associated with anti-atherogenic activities in vivo [28,29].

The anti-inflammatory actions of RSV extended beyond the atherosclerotic plaques to the peripheral blood (Table 1). Thus, RSV produced a reduction in B cells together with CD3+ T cells and CD8+ T subsets (Table 1). CD3+ T cells account for the second majority of leukocytes after monocytes/macrophages in mouse and human atherosclerotic plaques [24], and hence their reduction will potentially protect against lesion development. In relation to CD8+ T cells, previous studies noted that depletion of CD8+ T lymphocytes in hyperlipidaemic ApoE^−/−^ and LDLr^−/−^ mice resulted in a reduction in both atherosclerotic plaque burden and macrophage accumulation in the plaque [30,31]. In contrast, the transfer of CD8+ T cells to ApoE^−/−^ mice contributes to necrotic core formation and vulnerable atherosclerotic plaques [30]. Interestingly, depletion of CD8+ T cells in hypercholesterolaemic LDLr^−/−^ mice results in a reduction in mature monocytes in the bone marrow and spleen [31]. This could also potentially contribute to the observed reduction in macrophage content in plaque and increased plaque stability.

Previous studies on the impact of RSV on weight gain, together with changes in weight of various organs and plasma lipid profiles, have produced inconsistent findings, and this probably reflects differences in the dose of RSV employed, duration of the intervention, and mode of administration, together with the model and the strain used [32,33,34]. For example, RSV showed no effect on lipid profiles in double ApoE^−/−^/LDLr^−/−^ mice and New Zealand rabbits, though reduction in atherosclerotic plaque burden was seen [32,33,34]. The RSV-mediated significant reduction in LDL/VLDL levels (Table 1) is a major anti-atherogenic action of this polyphenol, and because this was not associated with changes in the plaque lipid content (Figure 1), this suggests that the major impact of this change is likely to be on inflammation and associated parameters (Figure 2 and Figure 3). No changes were seen in TG and HDL-C levels (Table 1), which is consistent with a recent meta-analysis in patients with metabolic syndrome [35]. Interestingly, IPA also predicted RSV-mediated modulation of cholesterol biosynthesis (Figure 7C). Thus, the expression of *acyl-CoA acyltransferase1/2* (*Acat1/2*), which is involved in the esterification and storage of cholesterol, and *3-hydroxy- 3methylgutaryl-CoA synthase* (*Hmgcs1*), involved in the biosynthesis of cholesterol [2], were significantly inhibited by RSV.

RNA-seq and subsequent downstream analysis also identified several genes and/or pathways that are potentially involved in the other anti-atherogenic actions of RSV seen in this study (Figure 7). The inhibition of mitochondrial oxidative phosphorylation pathway by RSV (Supplementary Figure S5) is likely to be a protective mechanism against ATP production and the activation of anabolic pathways produced by HFD and is also consistent with RSV-mediated inhibition of mitochondrial superoxide production (Figure 4E). Related to this was the predicted activation of the sirtuin signalling pathway, which serves as “metabolic sensors” that depends on the availability of NAD+ for activation and, in addition to enhancing metabolic efficiency, improves mitochondrial function [36,37]. This is consistent with the RSV-mediated inhibition of cellular ROS production (Figure 4 and Figure 5), which then positively impacts inflammation, lipid metabolism, and EC dysfunction.

RSV mediated several anti-inflammatory actions in vitro and in vivo, and indeed, RNA-seq revealed regulation of several pathways involved in the control of inflammatory responses. The crucial role of NF-κB in the initiation and progression of atherosclerosis, either directly or indirectly, is widely known [38], and RSV was predicted to inhibit its action (Supplementary Figure S6). Activated NF-κB is present in human atherosclerotic plaques, and modulation of its activity limits disease progression in ApoE knockout mice and produces a more stable plaque phenotype [38]. The NF-κB signalling pathway is also required for the induction of T cell signalling, T cell activation, and differentiation [39]; consistent with reduced plaque T cell content (Figure 2), this pathway was predicted to be inhibited by RSV (Supplementary Figure S7). Interestingly, inhibition of T cell activation has been shown in DBA/1J mice after RSV intervention that consequently prevents autoimmune disease progression [40]. In the context of atherosclerosis, ApoE^−/−^ mice fed HFD with lipopolysaccharide (LPS) (as an injection) and RSV (daily intragastric administration) inhibited the proliferation and activation of CD4+ T cells [41]. Consistent with the inhibition of cholesterol crystals-induced production of IL-1β (Figure 4G), the NLRP3 inflammasome signalling pathway was predicted to be inhibited by RSV (Supplementary Figure S8). The role of the NLRP3 inflammasome pathway in atherosclerosis is not fully understood, though it is important to note that this pathway is inhibited by colchicine, which is emerging as an important anti-inflammatory therapy against ACVD [2].

In addition to the inhibition of the pro-inflammatory pathways detailed above, the activation of sirtuins, which are known to inhibit NF-κB and inflammasome pathways [36], is likely to contribute to the anti-inflammatory actions of RSV. Furthermore, the activation of oxytocin signalling is known for its protective role via reducing inflammation and activation of oestrogen receptor signalling that then attenuates lipid accumulation and inflammation in female, as well as male ApoE and LDLr deficient mice [42]. The Wnt/β-catenin pathway was also predicated to be activated; however, in contrast to cancer, the role of this pathway in atherosclerosis is not fully understood [43]. Nevertheless, such activation could be due to the predicted inhibition of the Dickkopf-1(*Dkk1*) gene, an inhibitor of Wnt signalling pathway [43]. Indeed, several clinical and pre-clinical studies have demonstrated the role of *Dkk1* in promoting inflammation, inducing plaque vulnerability, and disease severity [43,44].

Metabolic pathways play important roles in inflammatory disorders, and RSV was predicted to inhibit glycolysis, ketogenesis, the TCA cycle, fatty acid β oxidation, and tryptophan degradation (Figure 7). Abnormalities in glycolysis flux accelerate atherosclerosis progression, and during inflammation, dysfunctional EC, macrophages, and migratory vascular SMC have high glycolytic capacity [45]. Ketogenesis is associated with heart failure, whilst reducing TCA cycle metabolites results in a decrease in NO and ROS levels in LPS-, TNFα-, or IFNγ-stimulated macrophages [46,47]. Increased rate of myocardial fatty acid β-oxidation in different rodent strains fed HFD is accompanied by heart failure, and switching to a low-fat diet or calorific restriction results in a reduction in fatty acid β-oxidation, and hence protection from heart failure [48]. Indeed, the ability of RSV to prevent or slow down the progression of heart failure in humans and animals has been reported previously [4,49]. The amino acid tryptophan is emerging as an important regulator of immune and inflammatory responses [50], and its degradation pathway was inhibited by RSV (Figure 7). IFN-γ produced by T-lymphocytes activates indoleamine 2,3-dioxygenase in immune cells, which in turn increases tryptophan catabolism into kynurenine and consequently increases serum kynurenine to tryptophan ratios and incidence of CVDs [50].

The plaque-stabilising action of RSV was a major anti-atherogenic action, and RSV significantly affected genes enriched in collagen synthesis and plaque stability (Figure 8C,D). Examples of genes implicated in plaque stability whose expression was induced by RSV included the *reversion-inducing-cysteine-rich protein with kazal motifs* (*Reck*) gene, implicated in the inhibition of enzymatic activities of MMPs [51]. In contrast, the expression of the *Rab interacting lysosomal protein* (*Rilp*) gene suggested as a marker for plaque instability [52] was downregulated. RSV was also predicted to inhibit the necroptosis signalling pathway, which has been linked to atherosclerosis and, in particular, plaque stability in animal and human studies [53]. Indeed, injection of hypercholesteraemic ApoE^−/−^ mice with a necroptotic inhibitor resulted in a reduction in both atherosclerotic plaque size and plaque instability markers, as well as attenuation of further progression of established lesions [53]. The reduced levels of ROS/mitochondrial ROS (Figure 4 and Figure 5) are also likely to contribute to plaque stability and correlates with the regulation of the antioxidant glutathione-mediated degradation pathway (Figure 7). In addition, increased expression of other genes such as *Ucp-2* (Uncoupling protein-2) is likely to contribute, given that its overexpression protects against mitochondrial dysfunction through a reduction in ROS production from this organelle, while bone marrow transplantation from *Ucp-2*^−/−^ mice into LDLr^−/−^ mice-supplemented with an atherogenic diet increased plaque content of macrophages and decreased collagen [54,55]. Many upstream regulators modulated by RSV also play important roles in atherosclerosis and plaque stability (Table 3). Thus, *Txnrd1*, whose expression was predicted to be inhibited by RSV codes for a key enzyme involved in cellular redox control and antioxidant defence mechanisms [56,57] (Supplementary Figure S10). The gene is expressed in atherosclerotic plaques, increased by oxidised LDL in HMDM, and its enhanced expression results in increased ROS production, NF-κB activity, and the release and expression of MCP-1 in human endothelial-like cells [56,57].

## 5. Conclusions

This study provides novel insights into the anti-atherogenic actions of RSV, together with the underlying molecular mechanisms. RSV attenuated plaque inflammation and produced a stable plaque phenotype. The anti-inflammatory actions of RSV in vivo extended to immune cells in the peripheral blood. In vitro studies provided additional mechanistic insights with RSV-mediated inhibition of ROS production, pro-inflammatory gene expression, chemokine-driven monocytic migration, and activation of the inflammasome. RNA-seq revealed key genes and pathways regulated by RSV that modulate its anti-atherogenic actions. Future studies should investigate whether the anti-inflammatory and plaque-stabilising actions of RSV extend to animal models of regression of existing/established atherosclerotic plaques and in clinical trials, and should examine the roles of identified key genes and pathways using knockdown or knockout approaches.

## Figures and Tables

**Table 1:** The effects of RSV on atherosclerosis-associated risk factors in male LDLr^−/−^ mice fed an HFD.

		HFD		HFD + RSV	
	N	Mean ± SEM	N	Mean ± SEM	*p*-Value or NS
***Overall weight gain *** **[g]**	30	7.92 ± 0.11	31	7.81 ± 0.07	NS
***Adipose tissue deposits *** **[g]**					
Total	20	0.055 ± 0.006	25	0.061 ± 0.003	NS
Total white	20	0.051 ± 0.006	25	0.057 ± 0.003	NS
Brown	20	0.004 ± 0.0003	25	0.004 ± 0.0002	NS
Subcutaneous	20	0.021 ± 0.002	24	0.019 ± 0.001	NS
Gonadal	21	0.026 ± 0.003	25	0.029 ± 0.002	NS
Inguinal	21	0.001 ± 0.0002	26	0.001 ± 0.0001	NS
Renal	21	0.008 ± 0.001	25	0.008 ± 0.001	NS
***Organ weights *** **[g]**					
Heart	30	0.005 ± 0.0002	31	0.004 ± 0.0002	0.054
Spleen	20	0.003 ± 0.0001	23	0.003 ± 0.0001	NS
Thymus	22	0.001 ± 0.0001	24	0.001 ± 0.0001	NS
Cardiac hypertrophy index [mg/mm]	20	9.22 ± 0.2	20	9.07 ± 0.2	NS
***Lipids *** **[mg/dL]**					
TG	18	88.9 ± 4.03	20	93.8 ± 8.32	NS
TC	13	1029.3 ± 38.70	20	925.7 ± 36.47	0.069
FC	17	593.9 ± 41.35	21	583.8 ± 30.02	NS
HDL-C	17	102.05 ± 14.98	21	115.5 ± 9.82	NS
LDL/VLDL-C	16	415.5 ± 17.74	19	319.3 ± 18.02	≤0.001
CE	18	693.7 ± 85.17	20	379.7 ± 34.5	≤0.001
***Peripheral blood—Lymphoid cells *** **(percentage of nucleated cells)**					
B cells	16	53.6 ± 1.5	22	42.3 ± 3.2	0.068
T cells	16	15.02 ± 0.47	22	13.2 ± 0.76	0.082
CD4 T cells	15	6.8 ± 0.25	21	6.0 ± 0.42	NS
CD8 T cells	16	7.0 ± 0.29	21	6.07 ± 0.38	0.089
NK cells	16	3.5 ± 0.24	21	3.9 ± 0.24	NS
***Peripheral blood—Myeloid cells *** **(percentage of nucleated cells)**					
Monocytes (CD115^+^)	18	6.8 ± 0.53	22	6.8 ± 0.27	NS
Ly6C^High^ monocytes	17	3.3 ± 0.32	22	3.5 ± 0.26	NS
Ly6C^Low^ monocytes	15	2.1 ± 0.15	24	2.2 ± 0.14	NS
Granulocytes	17	11.2 ± 1.15	22	14.7 ± 2.37	NS

CE—Cholesteryl esters; FC—free cholesterol; HDL-C—high-density lipoprotein-cholesterol; LDL/VLDL-C—low-density lipoprotein/very-low-density lipoprotein-cholesterol; NK—natural killer; NS—not significant; TC—total cholesterol; TG—triacylglycerol.

**Table 2:** List of top 20 upregulated DEGs by RSV intervention.

Gene	Log2 Fold Change	Adjusted *p*-Value (*p*adj.)	Function(s) of the Encoded Protein
*Sec14l3*	13.732	4.54 × 10^−36^	Has tumour-suppressive role
*Scgb1a1*	11.735	1.52 × 10^−26^	Modulates immune and inflammatory responses in alveolar macrophages and lungs
*Bpifa1*	11.378	5.76 × 10^−11^	Immunomodulatory properties in the context of airway inflammation
*Ccdc153*	11.182	3.12 × 10^−28^	Specific marker for ependymal cells
*Bpifb1*	11.161	8.04 × 10^−38^	Contributes to innate immune response
*Gabrp*	11.049	1.01 × 10^−21^	Inhibitory neurotransmitter in brain
*Muc5b*	10.890	1.52 × 10^−36^	Protective function in normal lung
*Scgb3a2*	10.795	1.74 × 10^−24^	Emerging growth factor in lungs
*Krt5*	10.658	2.29 × 10^−24^	Involved in structural framework of the skin
*Sftpa1*	10.363	5.70 × 10^−24^	Involved in lung homeostasis and defence against respiratory diseases
*Sftpd*	10.312	2.81 × 10^−23^	Involved in innate immune responses to protect lungs
*Cyp2a5*	10.209	2.72 × 10^−27^	Multiple roles, including regulation of enzyme activity
*5330417C22Rik*	10.184	7.97 × 10^−18^	Not known
*Cfap65*	10.161	1.29 × 10^−22^	Involved in spermiogenesis
*Slc5a8*	9.782	6.05 × 10^−20^	Involved in transport of molecules
*Tmem212*	9.750	4.21 × 10^−20^	Contributes to innate architecture of face processing
*Scgb3a1*	9.710	9.60 × 10^−21^	Anti-inflammatory and immunomodulatory actions in airway diseases
*Krt15*	9.664	4.28 × 10^−25^	Biomarker of epidermal stem cells
*Spag16*	9.632	4.78 × 10^−20^	Essential role in normal spermatogenesis and sperm motility
*Esrp1*	9.581	3.01 × 10^−18^	RNA-binding protein that regulates epithelial cells

Abbreviations: *Bpifa1*—BPI fold-containing family A, member 1; *Bpifb1*—BPI fold-containing family B member 1; *Ccdc153*—Coiled-coil domain-containing 153; *Cfap65*—Cilia- and flagella-associated protein 65; *Cyp2a5*—Cytochrome P450, family 2, subfamily a, polypeptide 5; *Esrp1*—Epithelial splicing regulatory protein 1; *Gabrp*—Gamma-aminobutyric acid type A receptor subunit pi; *Krt5*—Keratin 5; *Krt15*, Keratin 15; *Muc5b*, Mucin 5b; *Scgb1a1*—Secretoglobin, family 1A, member 1; *Scgb3a1—*Secretoglobin family 3A member 1; *Scgb3a2*—Secretoglobin family 3A member 2; *Sec14l3*—SEC14-like lipid-binding; *Sftpa1*—Pulmonary surfactant-associated protein A1; *Slc5a8*—Sodium-coupled monocarboxylate transporter 1; *Sftpd*—Pulmonary surfactant-associated protein D; *Spag16*—Sperm-associated antigen 16; *Tmem212*—Transmembrane protein 212.

**Table 3:** List of top 20 downregulated DEGs by RSV intervention.

Gene	Log2 Fold Change	Adjusted *p*-Value (*p*adj.)	Function(s) of the Encoded Protein
*Gm9694*	−3.776	0.029	Not known
*Gm44005*	−3.766	0.036	Not known
*Cntnap3*	−3.637	0.045	Regulates neuronal–glial and glial–glial interactions
*Adam4*	−3.625	0.020	Regulates cell phenotypes by controlling cell adhesion, migration, proteolysis, and signalling
*Gm39929*	−2.859	0.022	Not known
*S100g*	−2.668	0.009	Vitamin D-dependent calcium-binding protein
*Ppp1r3g*	−2.654	0.033	Regulates glucose homeostasis
*Mfsd2a*	−2.454	0.002	Sodium-dependent lysophosphatidylcholine transporter
*Entpd4b*	−2.393	0.037	Member of the apyrase protein family that catalyses the hydrolysis of nucleotide diphosphates and triphosphates
*Gm10790*	−2.360	0.011	Not known
*Ctse*	−2.289	0.043	Member of the A1 family of peptidases
*Gm43500*	−2.284	0.008	Not known
*1700061l17Rik*	−2.243	0.011	Not known
*Gm42814*	−2.209	0.002	Not known
*Sgk2*	−2.196	0.002	Mediates phosphorylation of proteins
*Gm45412*	−2.162	0.001	Not known
*Rdh16*	−2.157	0.018	Involved in steroid metabolic process
*Gm32468*	−2.124	1.48 × 10^−6^	Not known
*Themis*	−2.092	0.045	Involved in T cell development
*Hoxc8*	−2.086	0.0001	Plays important role in morphogenesis

Abbreviations: *Adam4*—A disintegrin and metallopeptidase domain 4; *Cntnap3*—Contactin-associated protein 3; *Ctse*—Cathepsin E; *Entpd4b*—Ectonucleoside triphosphate diphosphohydrolase 4; *Hoxc8*—Homeobox protein Hox-C8; *Mfsd2a*—MFSD2 Lysolipid transporter A, lysophospholipid; *Ppp1r3g*—Protein phosphatase 1 regulatory subunit 3G; *Rdh16*—Retinol Dehydrogenase 16; *Sgk2*—Serum/glucorticoid-regulated kinase 2; *S100g*—S100 calcium-binding protein G; *Themis*—Thymocyte-expressed molecule involved in selection-1.

**Table 4:** Top predicted upstream regulator genes affected by RSV intervention.

Upstream Regulator Gene	Function of Encoded Protein	Predicted Activation State	Activation z-Score	*p*-Value of Overlap
*Tead1*	Transcriptional regulator	Inhibited	−6.745	1.52 × 10^−28^
*Kdm5a*	Transcriptional regulator	Activated	3.976	2.03 × 10^−25^
*Map4k4*	Kinase	Activated	6.134	3.66 × 10^−24^
*Clpp*	Peptidase	Activated	6.733	3.79 × 10^−21^
*Tp53*	Transcriptional regulator	Activated	5.349	1.32 × 10^−18^
*Cpt1b*	Enzyme	Activated	6.589	7.92 × 10^−18^
*Slc27a2*	Transporter	Activated	5.274	6.07 × 10^−16^
*Insr*	Kinase	Inhibited	−5.383	2.70 × 10^−13^
*Esrra*	Transcriptional regulator	Inhibited	−2.161	1.31 × 10^−12^
*Ppargc1b*	Transcriptional regulator	Inhibited	−4.542	9.60 × 10^−12^
*Hba1/hba2*	Transporter	Inhibited	−3.536	1.01 × 10^−11^
*Klf15*	Transcriptional regulator	Inhibited	−4.027	1.06 × 10^−11^
*Nr4a1*	Ligand-dependent nuclear receptor	Activated	3.442	1.12 × 10^−10^
*Nrip1*	Transcriptional regulator	Activated	4.162	1.86 × 10^−10^
*Dmd*	Other	Activated	2.296	7.48 × 10^−10^
*Eif6*	Translation regulator	Activated	2.079	2.78 × 10^−9^
*Nrf1*	Transcriptional regulator	Inhibited	−2.683	2.99 × 10^−9^
*Ppargc1a*	Transcriptional regulator	Inhibited	−5.451	3.09 × 10^−9^
*Pitx2*	Transcriptional regulator	Inhibited	−2.498	8.39 × 10^−9^
*Por*	Enzyme	Activated	2.831	1.34 × 10^−8^
*Rictor*	Other	Activated	8.040	2.38 × 10^−8^
*Med13*	Transcriptional regulator	Activated	2.344	3.82 × 10^−8^
*Ctnnb1*	Transcriptional regulator	Activated	3.998	5.45 × 10^−8^
*Nampt*	Cytokine	Inhibited	−4.838	8.80 × 10^−8^
*Flcn*	Other	Activated	4.250	1.57 × 10^−7^
*Trib1*	Kinase	Inhibited	−3.514	2.23 × 10^−7^
*Stk11*	Kinase	Inhibited	−5.896	4.13 × 10^−7^
*Ppara*	Ligand-dependent nuclear receptor	Inhibited	−5.372	4.92 × 10^−7^
*Ccnc*	Other	Inhibited	−4.155	8.70 × 10^−7^
*Cidec*	Other	Activated	3.296	9.43 × 10^−7^
*Fgf21*	Growth factor	Inhibited	−3.322	9.52 × 10^−7^
*Ucp1*	Transporter	Inhibited	−4.473	1.77 × 10^−6^
*Asxl1*	Transcriptional regulator	Activated	2.982	1.80 × 10^−6^
*Nedd9*	Other	Inhibited	−4.536	3.76 × 10^−6^
*Txnrd1*	Enzyme	Inhibited	−2.242	3.83 × 10^−6^
*Gsr*	Enzyme	Inhibited	−2.198	3.83 × 10^−6^
*Pparg*	Ligand-dependent nuclear receptor	Inhibited	−5.597	8 × 10^−6^
*Irs1*	Enzyme	Inhibited	−2.895	8 × 10^−6^
*Ehhadh*	Enzyme	Activated	3.450	1.08 × 10^−5^

*Asxl1*—ASXL transcriptional regulator 1; *Ccnc*—Cyclin C; *Cidec*—Cell death-inducing DFFA-like effector C; *Clpp*—Caseinolytic mitochondrial matrix peptidase proteolytic subunit; *Cpt1b*—Carnitine palmitoyltransferase 1b; *Ctnnb1*—Catenin beta-1; *Dmd*—Dystrophin; *Ehhadh*—Enoyl Co-A hydratase and 3-hydroxyacyl CoA dehydrogenase; *Eif6*—Eukaryotic translation initiation factor 6; *Esrra*—Oestrogen-related receptor alpha; *Fgf21*—Fibroblast growth factor 21; *Flcn*—Folliculin; *Gsr*—Glutathione-disulfide reductase; *Hba1/hba2*—Haemoglobin subunit alpha 1/2; *Insr*—Insulin receptor; *Irs1*—Insulin receptor substrate 1; *Kdm5a*—Lysine Demethylase 5A; *Klf15*—Küppel-like factor 15; *Map4k4*—Mitogen-activated protein kinase kinase kinase kinase 4; *Med13*—Mediator complex subunit 13; *Nampt*—Nicotinamide phosphoribosyltransferase; *Nedd9*—Neural precursor cell expressed, developmentally downregulated 9; *Nr4a1*—Nuclear receptor subfamily 4 group a member 1; *Nrf1*—Nuclear respiratory factor 1; *Nrip1*—Nuclear receptor interacting protein 1; *Pitx2*—Paired-like homeodomain transcription factor 2; *Por*—Cytochrome P450 oxidoreductase; *Ppara*—Peroxisome proliferator-activated receptor alpha; *Pparg*—Peroxisome proliferator-activated receptor gamma; *Ppargc1a*—Peroxisome proliferator-activated receptor gamma coactivator 1-alpha; *Ppargc1b*—Peroxisome proliferator-activated receptor gamma coactivator 1-beta; *Rictor*—RPTOR independent companion of MTOR complex 2; *Slc27a2*—Solute carrier family 27 member 2; *Stk11*—Serine/threonine kinase 11; *Tead1*—TEA domain family member 1; *Tp53*—Tumour protein p53; *Trib1*—Tribbles pseudokinase 1; *Txnrd1*—Thioredoxin reductase; *UCP1*, Uncoupling protein 1.

## Data Availability

The original contributions presented in this study are included in the article and Supplementary Materials. Further inquiries can be directed to the corresponding author.

## Supplementary Materials

The following supporting information can be downloaded at https://www.mdpi.com/article/10.3390/antiox15010076/s1.

## Abbreviations

The following abbreviations are used in this manuscript:

Acat1/2	*Acyl-CoA acyltransferase1/2*
ACVD	Atherosclerotic cardiovascular disease
Adam4	A disintegrin and metallopeptidase domain 4
ANOVA	One-way analysis of variance
APC	Allophycocyanin
ApoE	Apolipoprotein E
Bfifa1	BPI fold containing family A, member 1
Bfifb1	BPI fold containing family B member 1
CE	Cholesteryl esters
Ctse	Cathepsin E
Cy7	Cyanine 7
DAPI	4′,6-diamidino-2-phenylindole, dilactate
DCFDA	2′,7′–dichorofluorescin diacetate
DEGs	Differentially Expressed Genes
Dkki	Dickkopf-1
ECM	Extracellular matrix
EC	Endothelial cells
Esrp1	Epithelial splicing regulatory protein 1
FC	Free cholesterol
FITC	Fluorescein isothiocyanate
GAPDH	Glyceraldehyde 3-phosphate dehydrogenase
GO	Gene Ontology
HAEC	Human aortic endothelial cells
HASMC	Human aortic smooth muscle cells
HDL-C	HDL-cholesterol
HFD	High-fat diet
HI-FCS	Heat-inactivated foetal calf serum
HMDM	Human monocyte-derived macrophages
Hmgcs1	3-hydroxy- 3methylgutaryl-CoA synthase
IFN-γ	Interferon-gamma
IL	Interleukin
IPA	Ingenuity Pathway Analysis
KEGG	Kyoto Encyclopaedia of Genes and Genomes
LDH	Lactate dehydrogenase
LDL	Low-density lipoprotein
LDL/VLDL-C	LDL/VLDL-Cholesterol
LDLr^−/−^	LDL receptor deficient
LPS	Lipopolysachharide
MCP-1	Monocyte chemotactic protein-1
Mfsd2a	MFSD2 Lysolipid transporter A
MitoSox	Mitochondrial Superoxide
MMP	Matrix metalloproteinases
NF-κB	Nuclear factor kappa-light-chain-enhancer of activated B cells
NLRP3	Nucleotide-binding domain, leucine-rich–containing family, pyrin domain–containing-3
NO	Nitric oxide
ORO	Oil Red O
*p*adj.	Adjusted *p*-value
PDGF	Platelet-derived growth factor
PE	Phycoerythrin
PerCP	Peridinin-Chlorophyll-Protein
Ppr1r3g	Protein phosphatase 1 regulatory subunit 3G
Reck	Reversion-inducing-cysteine-rich protein with kazal motifs
Rilp	Rab interacting lysosomal protein
RNA-seq	RNA-sequencing
ROS	Reactive oxygen species
RSV	Resveratrol
Scgb1a1	Secretoglobin, family 1A, member 1
Scgb3a1	Secretoglobin, family 3A, member 1
Sftpd	Pulmonary surfactant-associated protein D
SMC	Smooth muscle cells
TBHP	Tert-butyl hydroperoxide
TC	Total cholesterol
TCA	Tricarboxylic acid
TG	Triacylglycerol
Themis	Thymocyte-expressed molecule involved in selection-1
TNF-α	Tumour necrosis factor-alpha
Txnrd1	Thioredoxin reductase 1
Ucp2	Uncoupling protein-2
Wnt	Wingless and int-1

## References

[1] Moss J.W., Ramji D.P. (2016). Nutraceutical therapies for atherosclerosis. Nat. Rev. Cardiol..

[2] Chan Y.H., Ramji D.P. (2022). Atherosclerosis: Pathogenesis and key cellular processes, current and emerging therapies, key challenges, and future research directions. Methods Mol. Biol..

[3] Raj P., Thandapilly S.J., Wigle J., Zieroth S., Netticadan T. (2021). A comprehensive analysis of the efficacy of resveratrol in atherosclerotic cardiovascular disease, myocardial infarction and heart failure. Molecules.

[4] Zhang L.X., Li C.X., Kakar M.U., Khan M.S., Wu P.F., Amir R.M., Dai D.F., Naveed M., Li Q.Y., Saeed M. (2021). Resveratrol (RV): A pharmacological review and call for further research. Biomed. Pharmacother..

[5] Cheng C.K., Luo J.Y., Lau C.W., Chen Z.Y., Tian X.Y., Huang Y. (2020). Pharmacological basis and new insights of resveratrol action in the cardiovascular system. Br. J. Pharmacol..

[6] Jing Y., Hu T., Yuan J., Liu Z., Tao M., Ou M., Cheng X., Cheng W., Yi Y., Xiong Q. (2023). Resveratrol protects against postmenopausal atherosclerosis progression through reducing PCSK9 expression via the regulation of the ERα-mediated signaling pathway. Biochem. Pharmacol..

[7] Ji W., Sun J., Hu Z., Sun B. (2022). Resveratrol protects against atherosclerosis by downregulating the PI3K/AKT/mTOR signaling pathway in atherosclerosis model mice. Exp. Ther. Med..

[8] Sirasanagandla S.R., Al-Huseini I., Al Mushaiqri M., Al-Abri N., Al-Ghafri F. (2022). Maternal resveratrol supplementation ameliorates bisphenol A-induced atherosclerotic lesions formation in adult offspring ApoE. 3 Biotech.

[9] Li J., Zhong Z., Yuan J., Chen X., Huang Z., Wu Z. (2019). Resveratrol improves endothelial dysfunction and attenuates atherogenesis in apolipoprotein E-deficient mice. J. Nutr. Biochem..

[10] Voloshyna I., Teboul I., Littlefield M.J., Siegart N.M., Turi G.K., Fazzari M.J., Carsons S.E., DeLeon J., Reiss A.B. (2016). Resveratrol counters systemic lupus erythematosus-associated atherogenicity by normalizing cholesterol efflux. Exp. Biol. Med..

[11] Ramji D.P., Chan Y.H., Alahmadi A., Alotibi R., Alshehri N. (2022). Survey of approaches for investigation of atherosclerosis in vivo. Methods Mol. Biol..

[12] Chassot L.N., Scolaro B., Roschel G.G., Cogliati B., Cavalcanti M.F., Abdalla D.S.P., Castro I.A. (2018). Comparison between red wine and isolated trans-resveratrol on the prevention and regression of atherosclerosis in LDLr. J. Nutr. Biochem..

[13] Lee Y.E., Kim J.W., Lee E.M., Ahn Y.B., Song K.H., Yoon K.H., Kim H.W., Park C.W., Li G., Liu Z. (2012). Chronic resveratrol treatment protects pancreatic islets against oxidative stress in db/db mice. PLoS ONE.

[14] Chang G.R., Chen P.L., Hou P.H., Mao F.C. (2015). Resveratrol protects against diet-induced atherosclerosis by reducing low-density lipoprotein cholesterol and inhibiting inflammation in apolipoprotein E-deficient mice. Iran. J. Basic Med. Sci..

[15] Zhuang Y., Huang H., Liu S., Liu F., Tu Q., Yin Y., He S. (2021). Resveratrol improves growth performance, intestinal morphology, and microbiota composition and metabolism in mice. Front. Microbiol..

[16] Chan Y.H., Moss J.W.E., Williams J.O., Ferekidis N., Alshehri N., Hughes T.R., Menendez-Gonzalez J.B., Plummer S.F., Michael D.R., Rodrigues N.P. (2023). (+)-catechin attenuates multiple atherosclerosis-associated processes in vitro, modulates disease-associated risk factors in C57BL/6J mice and reduces atherogenesis in LDL receptor deficient mice by inhibiting inflammation and increasing markers of plaque stability. Mol. Nutr. Food Res..

[17] O’Morain V.L., Chan Y.H., Williams J.O., Alotibi R., Alahmadi A., Rodrigues N.P., Plummer S.F., Hughes T.R., Michael D.R., Ramji D.P. (2021). The Lab4P consortium of probiotics attenuates atherosclerosis in LDL receptor deficient mice fed a high fat diet and causes plaque stabilization by inhibiting inflammation and several pro-atherogenic processes. Mol. Nutr. Food Res..

[18] Al-Ahmadi W., Webberley T.S., Joseph A., Harris F., Chan Y.H., Alotibi R., Williams J.O., Alahmadi A., Decker T., Hughes T.R. (2021). Pro-atherogenic actions of signal transducer and activator of transcription 1 serine 727 phosphorylation in LDL receptor deficient mice via modulation of plaque inflammation. FASEB J..

[19] Nair A.B., Jacob S. (2016). A simple practice guide for dose conversion between animals and human. J. Basic Clin. Pharm..

[20] Reagan-Shaw S., Nihal M., Ahmad N. (2008). Dose translation from animal to human studies revisited. FASEB J..

[21] Moss J.W.E., Williams J.O., Al-Ahmadi W., O’Morain V., Chan Y.H., Hughes T.R., Menendez-Gonzalez J.B., Almotiri A., Plummer S.F., Rodrigues N.P. (2021). Protective effects of a unique combination of nutritionally active ingredients on risk factors and gene expression associated with atherosclerosis in C57BL/6J mice fed a high fat diet. Food Funct..

[22] O’Morain V.L., Chen J., Plummer S.F., Michael D.R., Ramji D.P. (2023). Anti-atherogenic actions of the Lab4b consortium of probiotics in vitro. Int. J. Mol. Sci..

[23] Qin Z. (2012). The use of THP-1 cells as a model for mimicking the function and regulation of monocytes and macrophages in the vasculature. Atherosclerosis.

[24] Ramji D.P., Davies T.S. (2015). Cytokines in atherosclerosis: Key players in all stages of disease and promising therapeutic targets. Cytokine Growth Factor Rev..

[25] Cojocaru K.A., Luchian I., Goriuc A., Antoci L.M., Ciobanu C.G., Popescu R., Vlad C.E., Blaj M., Foia L.G. (2023). Mitochondrial dysfunction, oxidative stress, and therapeutic strategies in diabetes, obesity, and cardiovascular disease. Antioxidants.

[26] Johnson J.L. (2017). Metalloproteinases in atherosclerosis. Eur. J. Pharmacol..

[27] Yalcinkaya M., Tall A.R. (2025). Cholesterol crystals as triggers of NLRP3 inflammasome activation in atherosclerosis. Curr. Atheroscler. Rep..

[28] He C., Medley S.C., Hu T., Hinsdale M.E., Lupu F., Virmani R., Olson L.E. (2015). PDGFRβ signalling regulates local inflammation and synergizes with hypercholesterolaemia to promote atherosclerosis. Nat. Commun..

[29] Ricci C., Ferri N. (2015). Naturally occurring PDGF receptor inhibitors with potential anti-atherosclerotic properties. Vasc. Pharmacol..

[30] Kyaw T., Winship A., Tay C., Kanellakis P., Hosseini H., Cao A., Li P., Tipping P., Bobik A., Toh B.H. (2013). Cytotoxic and proinflammatory CD8+ T lymphocytes promote development of vulnerable atherosclerotic plaques in apoE-deficient mice. Circulation.

[31] Cochain C., Koch M., Chaudhari S.M., Busch M., Pelisek J., Boon L., Zernecke A. (2015). CD8+ T cells regulate monopoiesis and circulating Ly6C-high monocyte levels in atherosclerosis in mice. Circ. Res..

[32] Wang Z., Huang Y., Zou J., Cao K., Xu Y., Wu J.M. (2002). Effects of red wine and wine polyphenol resveratrol on platelet aggregation in vivo and in vitro. Int. J. Mol. Med..

[33] Wang Z., Zou J., Cao K., Hsieh T.C., Huang Y., Wu J.M. (2005). Dealcoholized red wine containing known amounts of resveratrol suppresses atherosclerosis in hypercholesterolemic rabbits without affecting plasma lipid levels. Int. J. Mol. Med..

[34] Fukao H., Ijiri Y., Miura M., Hashimoto M., Yamashita T., Fukunaga C., Oiwa K., Kawai Y., Suwa M., Yamamoto J. (2004). Effect of trans-resveratrol on the thrombogenicity and atherogenicity in apolipoprotein E-deficient and low-density lipoprotein receptor-deficient mice. Blood Coagul. Fibrinolysis.

[35] Akbari M., Tamtaji O.R., Lankarani K.B., Tabrizi R., Dadgostar E., Haghighat N., Kolahdooz F., Ghaderi A., Mansournia M.A., Asemi Z. (2020). The effects of resveratrol on lipid profiles and liver enzymes in patients with metabolic syndrome and related disorders: A systematic review and meta-analysis of randomized controlled trials. Lipids Health Dis..

[36] Akantibila M., Carabetta V.J. (2025). Sirtuins as therapeutic targets for treating cancer, metabolic diseases, and neurodegenerative diseases. Pharmaceuticals.

[37] Houtkooper R.H., Pirinen E., Auwerx J. (2012). Sirtuins as regulators of metabolism and healthspan. Nat. Rev. Mol. Cell Biol..

[38] Mallavia B., Recio C., Oguiza A., Ortiz-Muñoz G., Lazaro I., Lopez-Parra V., Lopez-Franco O., Schindler S., Depping R., Egido J. (2013). Peptide inhibitor of NF-κB translocation ameliorates experimental atherosclerosis. Am. J. Pathol..

[39] Liu T., Zhang L., Joo D., Sun S.C. (2017). NF-κB signaling in inflammation. Signal Transduct. Target. Ther..

[40] Xuzhu G., Komai-Koma M., Leung B.P., Howe H.S., McSharry C., McInnes I.B., Xu D. (2012). Resveratrol modulates murine collagen-induced arthritis by inhibiting Th17 and B-cell function. Ann. Rheum. Dis..

[41] Zhou L., Long J., Sun Y., Chen W., Qiu R., Yuan D. (2020). Resveratrol ameliorates atherosclerosis induced by high-fat diet and LPS in ApoE. Nutr. Metab..

[42] Nofer J.R. (2012). Estrogens and atherosclerosis: Insights from animal models and cell systems. J. Mol. Endocrinol..

[43] Afroz R., Goodwin J.E. (2024). Wnt signaling in atherosclerosis: Mechanisms to therapeutic implications. Biomedicines.

[44] Di M., Wang L., Li M., Zhang Y., Liu X., Zeng R., Wang H., Chen Y., Chen W., Zhang M. (2017). Dickkopf1 destabilizes atherosclerotic plaques and promotes plaque formation by inducing apoptosis of endothelial cells through activation of ER stress. Cell Death Dis..

[45] Li L., Wang M., Ma Q., Ye J., Sun G. (2022). Role of glycolysis in the development of atherosclerosis. Am. J. Physiol. Cell Physiol..

[46] Cotter D.G., Schugar R.C., Crawford P.A. (2013). Ketone body metabolism and cardiovascular disease. Am. J. Physiol. Heart Circ. Physiol..

[47] Liang Y., Chen Y., Li L., Zhang S., Xiao J., Wei D. (2022). Krebs cycle rewired: Driver of atherosclerosis progression?. Curr. Med. Chem..

[48] Fillmore N., Mori J., Lopaschuk G.D. (2014). Mitochondrial fatty acid oxidation alterations in heart failure, ischaemic heart disease and diabetic cardiomyopathy. Br. J. Pharmacol..

[49] Zhang L., Chen J., Yan L., He Q., Xie H., Chen M. (2021). Resveratrol ameliorates cardiac remodeling in a murine model of heart failure with preserved ejection fraction. Front. Pharmacol..

[50] Sudar-Milovanovic E., Gluvic Z., Obradovic M., Zaric B., Isenovic E.R. (2022). Tryptophan metabolism in atherosclerosis and diabetes. Curr. Med. Chem..

[51] Meng N., Li Y., Zhang H., Sun X.F. (2008). RECK, a novel matrix metalloproteinase regulator. Histol. Histopathol..

[52] Xu B.F., Liu R., Huang C.X., He B.S., Li G.Y., Sun H.S., Feng Z.P., Bao M.H. (2020). Identification of key genes in ruptured atherosclerotic plaques by weighted gene correlation network analysis. Sci. Rep..

[53] Karunakaran D., Geoffrion M., Wei L., Gan W., Richards L., Shangari P., DeKemp E.M., Beanlands R.A., Perisic L., Maegdefessel L. (2016). Targeting macrophage necroptosis for therapeutic and diagnostic interventions in atherosclerosis. Sci. Adv..

[54] Tian X.Y., Ma S., Tse G., Wong W.T., Huang Y. (2018). Uncoupling protein 2 in cardiovascular health and disease. Front. Physiol..

[55] Blanc J., Alves-Guerra M.C., Esposito B., Rousset S., Gourdy P., Ricquier D., Tedgui A., Miroux B., Mallat Z. (2003). Protective role of uncoupling protein 2 in atherosclerosis. Circulation.

[56] Furman C., Rundlöf A.K., Larigauderie G., Jaye M., Bricca G., Copin C., Kandoussi A.M., Fruchart J.C., Arnér E.S., Rouis M. (2004). Thioredoxin reductase 1 is upregulated in atherosclerotic plaques: Specific induction of the promoter in human macrophages by oxidized low-density lipoproteins. Free Radic. Biol. Med..

[57] Liu Z.B., Shen X. (2009). Thioredoxin reductase 1 upregulates MCP-1 release in human endothelial cells. Biochem. Biophys. Res. Commun..

