# Supplementary material for: Resveratrol Mediates Anti-Atherogenic Actions In Vitro and in LDL Receptor-Deficient Mice Fed a High-Fat Diet via Antioxidant, Anti-Inflammatory and Plaque-Stabilising Activities
Source: Antioxidants (Basel). 2026 Jan 7;15(1):76. doi: 10.3390/antiox15010076 (PMC12837184; doi:10.3390/antiox15010076)
Supplement: Supplementary file 1 [file antioxidants-15-00076-s001.zip › antioxidants-4022176-supplementary.pdf]

Resveratrol Mediates Anti-Atherogenic Actions In Vitro and in LDL Receptor-Deficient Mice Fed a High-Fat Diet via Antioxidant, Anti-Inflammatory and Plaque-Stabilising Activities

by

Alaa Alahmadi[1,2], Reem Alotibi[1,2], Yee-Hung Chan[1], Sarab Taha[3], Daniah Rifqi[1,] Nouf Alshehri[1,2], Sulaiman Alalawi[1], Fahad Alradi[1], Alex Gibbs[3], Timothy R. Hughes[4] and Dipak P. Ramji[1*]

[1]Cardiff School of Biosciences, Cardiff University, Sir Martin Evans Building, Museum Avenue, Cardiff CF10 3AX, UK.

[2] Department of Biological Science, College of Science, University of Jeddah, Jeddah 21589, Saudi Arabia.

[3]European Cancer Stem Cell Research Institute, Cardiff School of Biosciences, Cardiff University, Hadyn Ellis Building, Maindy Road, Cardiff CF24 4HQ, UK.

[4]Division of Infection and Immunity, Henry Wellcome Building, School of Medicine, Cardiff University, Heath Park, Cardiff CF14 4XN, UK.

***Corresponding author:** Professor Dipak P. Ramji, Cardiff School of Biosciences, Cardiff University, Sir Martin Evans Building, Museum Avenue, Cardiff CF10 3AX, UK. Tel: 0044 (0)29 20876753; Email: Ramji@Cardiff.ac.uk

Supplementary Table S1: Antibodies used for flow cytometry

Reagent	Fluorochrome where applicable	Supplier	Clone	Catalogue number
CD115	PE	BioLegend	AFS98	135505
Ly6C	PE-Cy7	BioLegend	HK1.4	128018
Ly6G	FITC	BioLegend	1A8	127605
CD3	PE	BioLegend	17A2	100206
CD4	PerCP	BioLegend	RM4-5	100537
CD8	APC-Cy7	BioLegend	53-6.7	100714
NK1.1	PE-Cy7	BioLegend	PK163	108714
B220	FITC	BioLegend	RA3-6B2	103206

FITC, fluorescein isothiocyanate; APC, allophycocyanin; PE, phycoerythrin; Cy7, cyanine-7; PerCP, peridinin-chlorophyll-protein

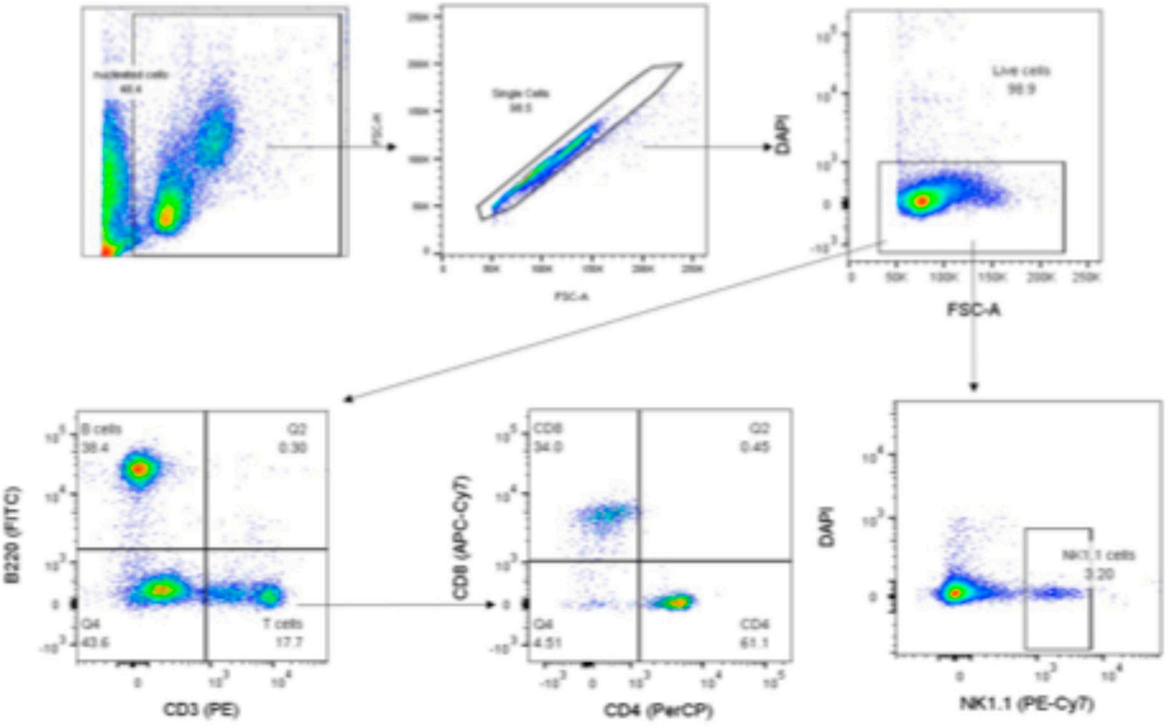

Supplementary Figure S1. Sequential plots for gating strategy for the identification of peripheral blood lymphoid cell populations by flow cytometry
The plots illustrate the gating strategy for the identification of the lymphoid cell populations in the peripheral blood.

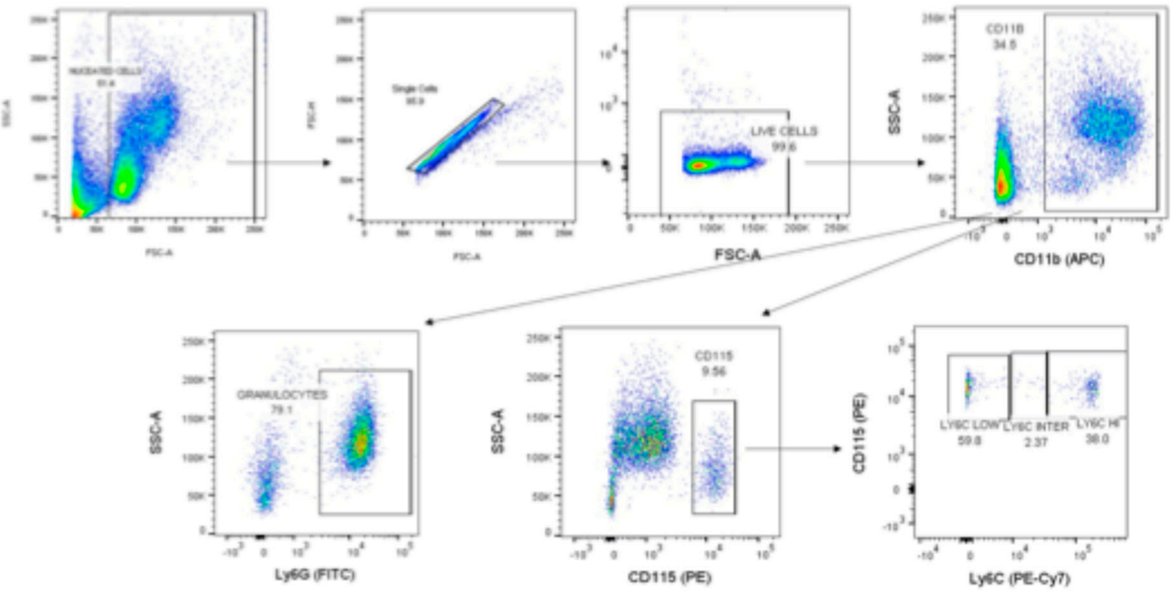

Supplementary Figure S2. Sequential plots for gating strategy for the identification of peripheral blood myeloid cell populations by flow cytometry
The plots illustrate the gating strategy for the identification of the myeloid cell populations in the peripheral blood.

(A) (B)

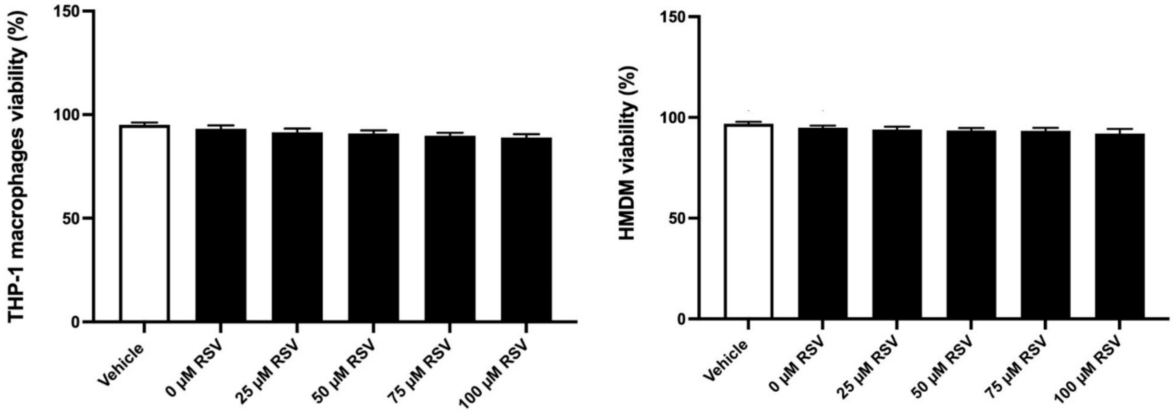

Supplementary Figure S3. RSV has no detrimental effects on cell viability of THP-1 and primary human monocyte-derived macrophages

THP-1 macrophages (**A**) and human monocyte-derived macrophages (HMDM; **B**) were treated with the vehicle control (DMSO) or the indicated concentration of RSV for 24 hours. Lactate dehydrogenase (LDH) assay kit was used on the supernatant to assess cell viability. Data (mean ± SEM from three or four independent experiments) are represented to the vehicle control that has been arbitrarily assigned as 100%. Statistical analysis was performed using one-way ANOVA with Dunnett (**A**) or Tukey's post-hoc analysis (**B**). No significant changes were observed.

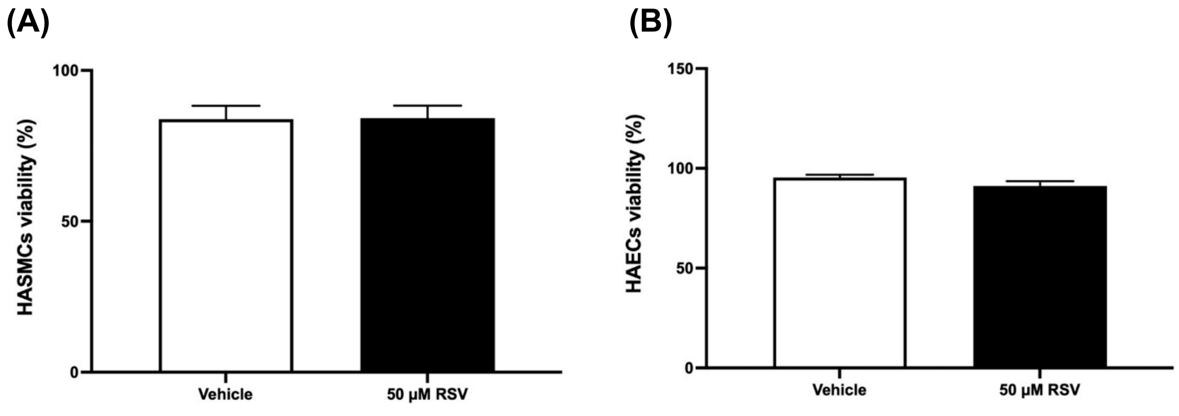

Supplementary Figure S4. RSV has no detrimental effects on cell viability of human aortic smooth muscle and endothelial cells

Human aortic smooth muscle cells (HASMCs; **A**) and human aortic endothelial cells (HAECs; **B**) were treated with the vehicle control (DMSO) or 50 μM RSV for 24 h. Cell viability was then assessed using the LDH assay kit. The results are presented as mean ± SEM relative to the vehicle control, which was set to 100% from three independent experiments. Statistical analysis was performed using an unpaired student's t-test. No significant changes were observed.

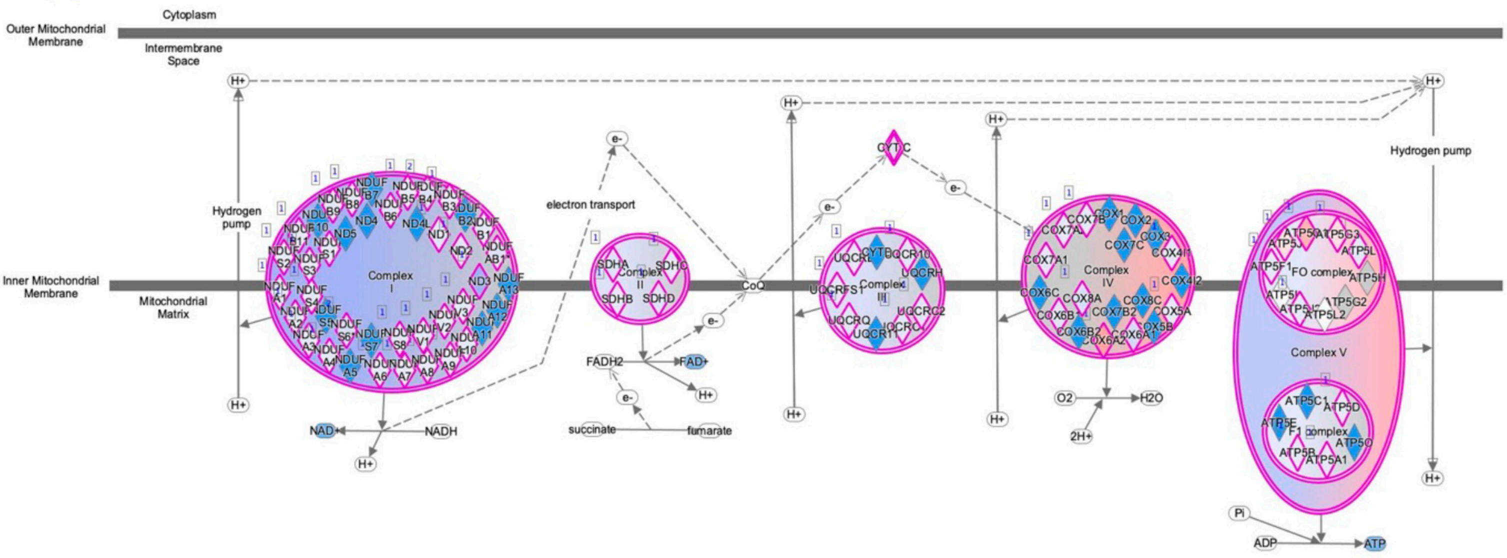

Supplementary Figure S5. Schematic representation of oxidative phosphorylation pathway (OXPHOS) affected by resveratrol treatment

The OXPHOS emerged as the most significantly enriched pathway inhibited by RSV. The inner mitochondrial membrane hosts five multi-subunit enzyme complexes (Complex I-V) and two electron carriers – coenzyme Q10 (CoQ10) and cytochrome C. The five complexes contain NADH: ubiquinone oxidoreductase (complex I), succinate: ubiquinone oxidoreductase (complex II), ubiquinol: ferrocytochrome c oxidoreductase (complex III), ferrocytochrome c: oxygen oxidoreductase or cytochrome c oxidase (complex IV), and ATP synthase (complex V). In the pathway, complex I catalyses the transfer of electrons from NADH to coenzyme Q (ubiquinone) that carries electrons from complex I to complex III. Complex II receives electrons from the TCA cycle intermediate, succinate, and catalyses the transport of electrons to FADH2 and then to coenzyme Q (ubiquinone), subsequently transferring electrons to complex III. In complex III, electrons transfer from ubiquinol to cytochrome C that brings electrons to complex IV. Finally, complex IV uses these electrons to reduce oxygen to water. The complex V is the final enzyme in the oxidative phosphorylation pathway and uses the energy stored in a proton gradient generated by respiratory chain to produce ATP from ADP. The genes in blue are those whose expression is suppressed while the genes in red are those whose expression is induced. Node intensity colour reflects the degree of significance whereases bright colour is less significant. Image generated by the IPA program.

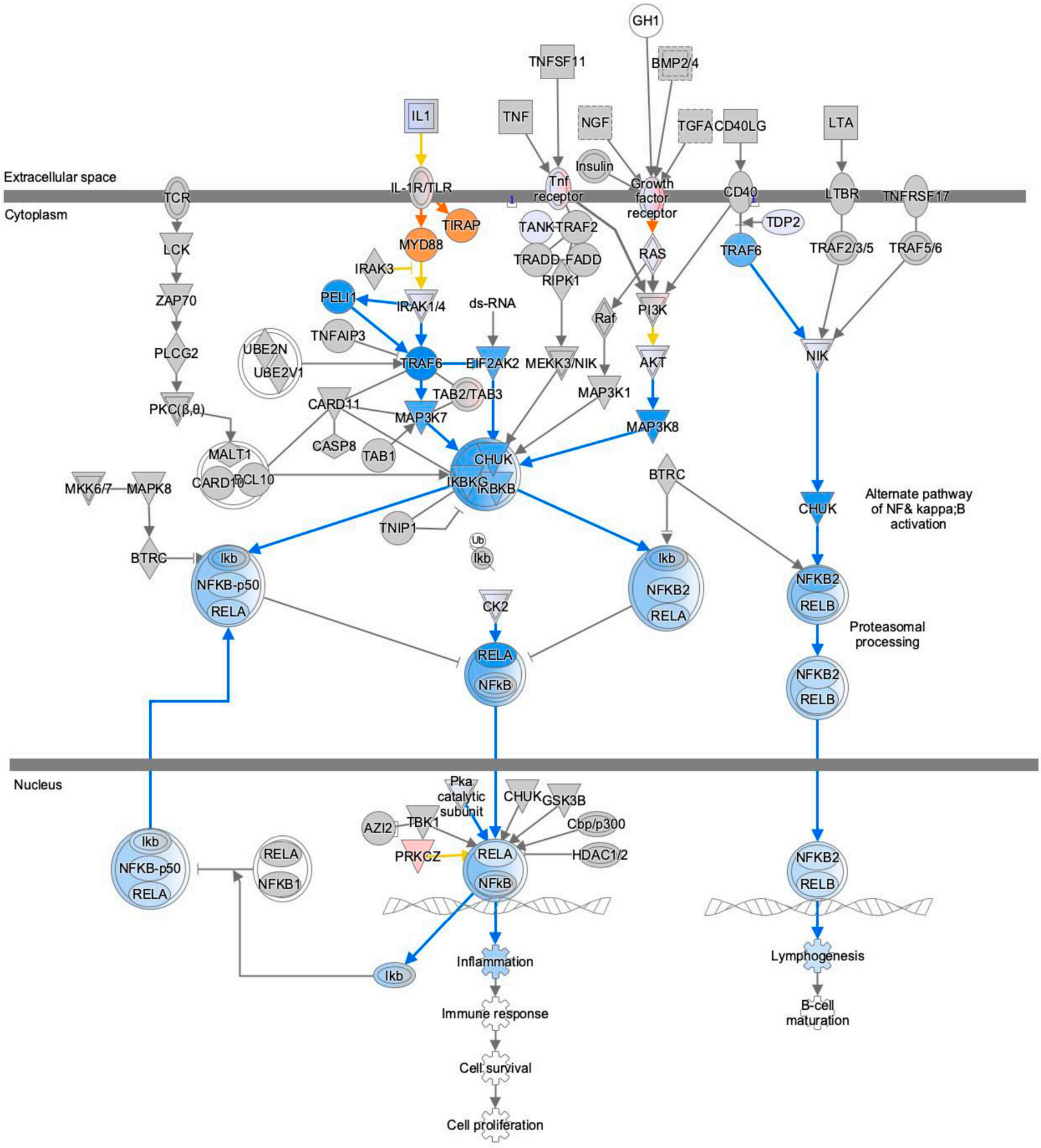

Supplementary Figure S6. The NF-κB pathway was predicted to be inhibited by RSV

The figure displays the NF-κB signalling pathway in which the genes in the data set were overlapped to visualise the effect of RSV intervention on genes implicated in the pathway. The nodes in blue represent down-regulated genes while the red/orange nodes represent up-regulated genes. Genes in blue and red/orange colour are in the data set and are significantly dysregulated ($p<0.05$) while genes in grey colour are also in the data set, but they are not significantly changed.

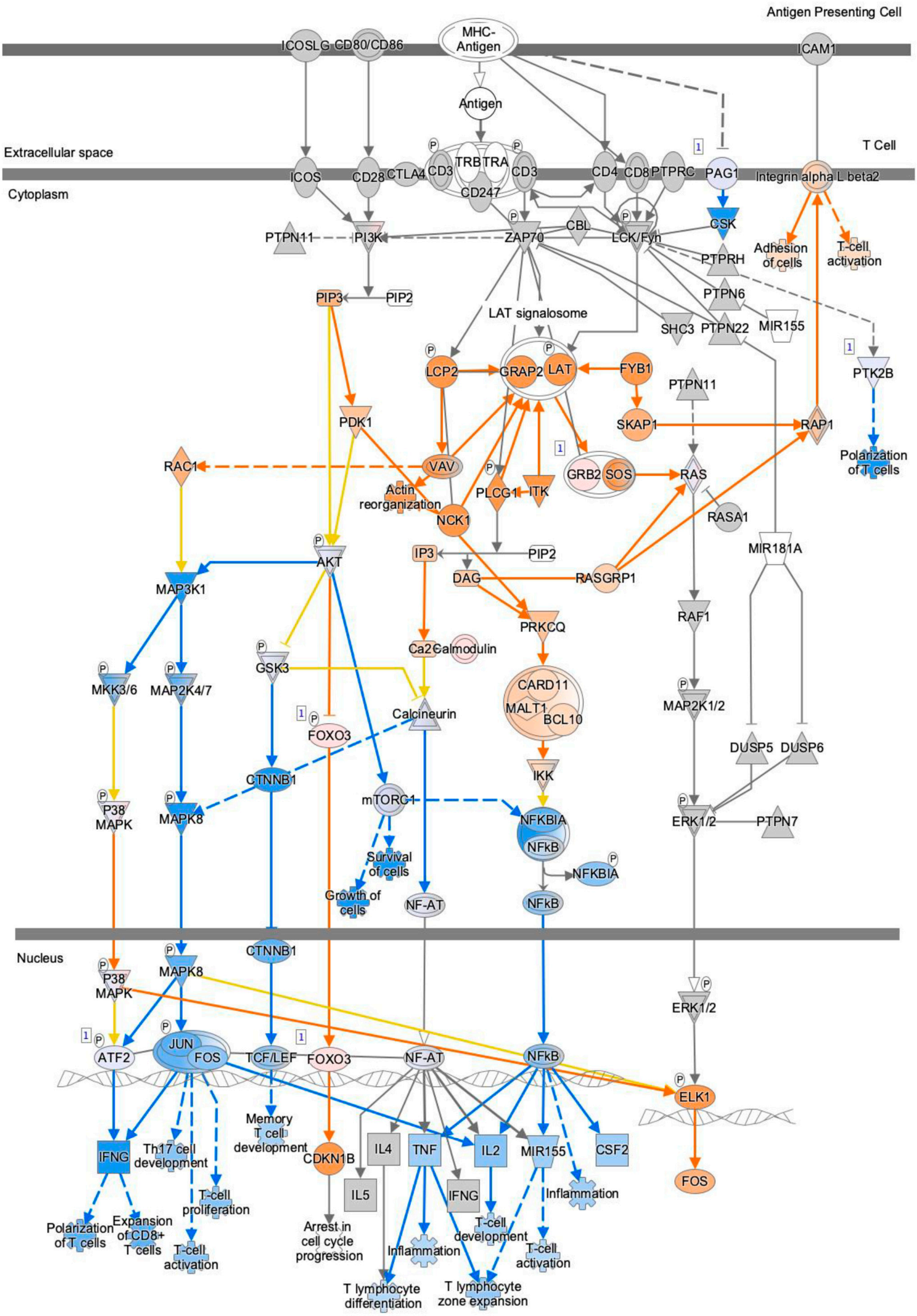
Antigen Presenting Cell
ICOSLG
CD80/CD86
MHC-Antigen
ICAM1
Antigen
Extracellular space
T Cell
Cytoplasm
ICOS
CD28
CTLA4
CD3
TRB
TRA
CD247
CD4
CD8
PTPRC
PAG1
Integrin alpha L beta2
PTPN11
PI3K
ZAP70
CBL
LCK/Fyn
CSK
Adhesion of cells
T-cell activation
PTPRH
PTPN6
PIP3
PIP2
LAT signalosome
SHC3
PTPN22
MIR155
PTK2B
LCP2
GRAP2
LAT
FYB1
PTPN11
PDK1
SKAP1
RAP1
Polarization of T cells
RAC1
VAV
GRB2
SOS
RAS
Actin reorganization
PLCG1
ITK
NCK1
RASA1
MIR181A
AKT
IP3
PIP2
DAG
RASGRP1
MAP3K1
RAF1
PRKCQ
Ca2
Calmodulin
GSK3
CARD11
MALT1
BCL10
MKK3/6
MAP2K4/7
MAP2K1/2
Calcineurin
FOXO3
DUSP5
DUSP6
IKK
CTNNB1
mTORC1
P38 MAPK
MAPK8
NFKBIA
ERK1/2
PTPN7
NFkB
Survival of cells
NFKBIA
Growth of cells
NF-AT
NFkB
Nucleus
CTNNB1
MAPK8
P38 MAPK
ERK1/2
ATF2
JUN
FOS
TCF/LEF
FOXO3
NF-AT
NFkB
ELK1
Memory T cell development
IFNG
Th17 cell development
IL4
TNF
IL2
MIR155
CSF2
CDKN1B
FOS
IL5
IFNG
Inflammation
T-cell proliferation
Polarization of T cells
Expansion of CD8+ T cells
T-cell activation
Arrest in cell cycle progression
Inflammation
T-cell development
T-cell activation
T lymphocyte differentiation
T lymphocyte zone expansion

Supplementary Figure S7. T cell signalling pathway was predicted to be inhibited by RSV

The figure displays T cell signalling pathway in which the genes in the data set were overlapped to visualise the effect of RSV intervention on genes implicated in the pathway. The nodes in blue represent down-regulated genes while the red/orange nodes represent up-regulated genes. Genes in blue and red/orange colour are in the data set and are significantly dysregulated ($p<0.05$) while genes in grey colour are also in the data set, but they are not significantly changed. The figure shows a reduction in inflammation, T cell polarisation and activation, and expansion of $CD8^+$ T cells.

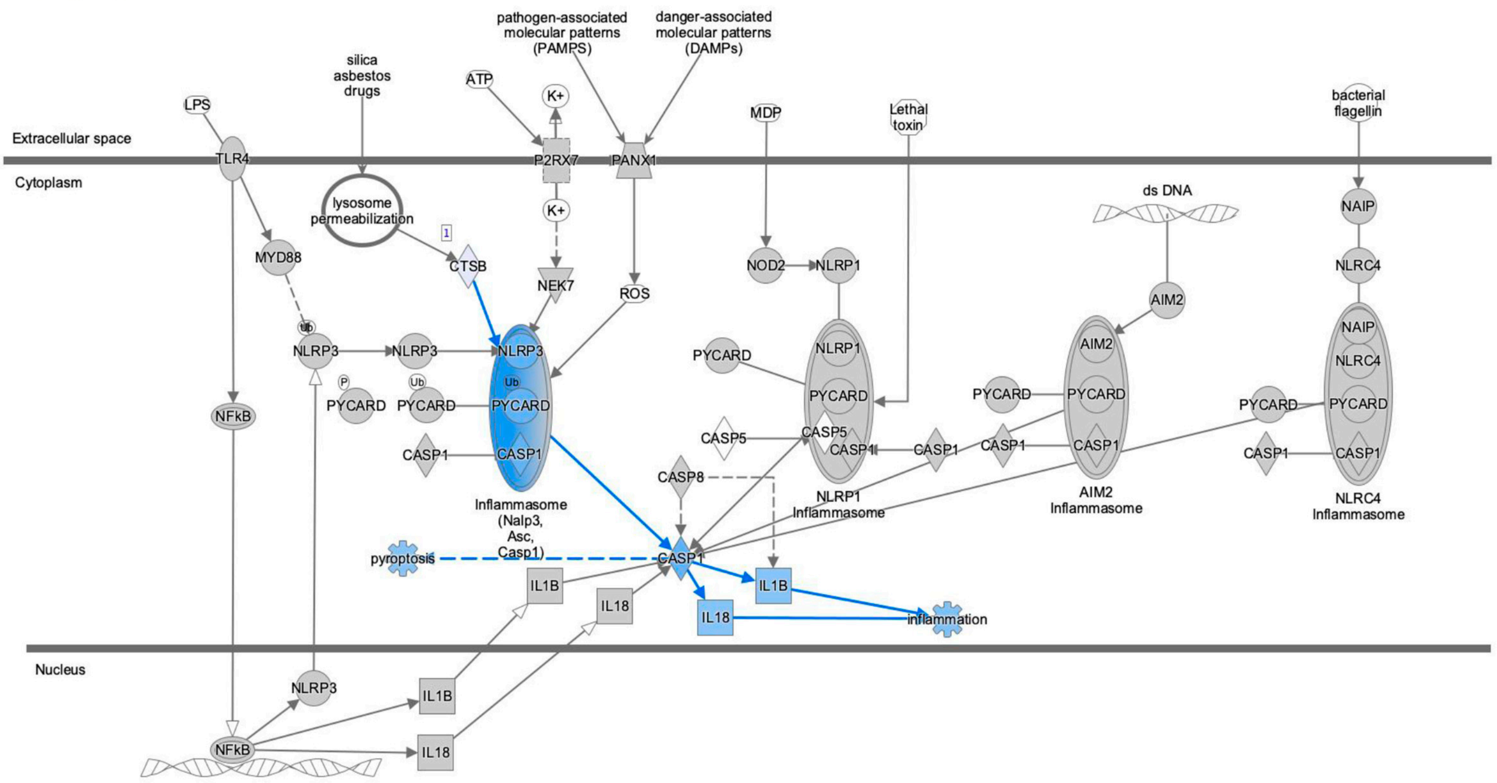

Supplementary Figure S8. NLRP3 inflammasome activation was predicted to be inhibited by RSV intervention

The figure displays the inflammasome activation pathway in which the genes in the data set were overlapped to visualise the effect of RSV intervention on genes implicated in the pathway. The nodes in blue represent down-regulated genes. Genes in blue nodes are in the data set, and they are significantly dysregulated ($p<0.05$) while genes in grey nodes are also in the data set, but they are not significantly changed.

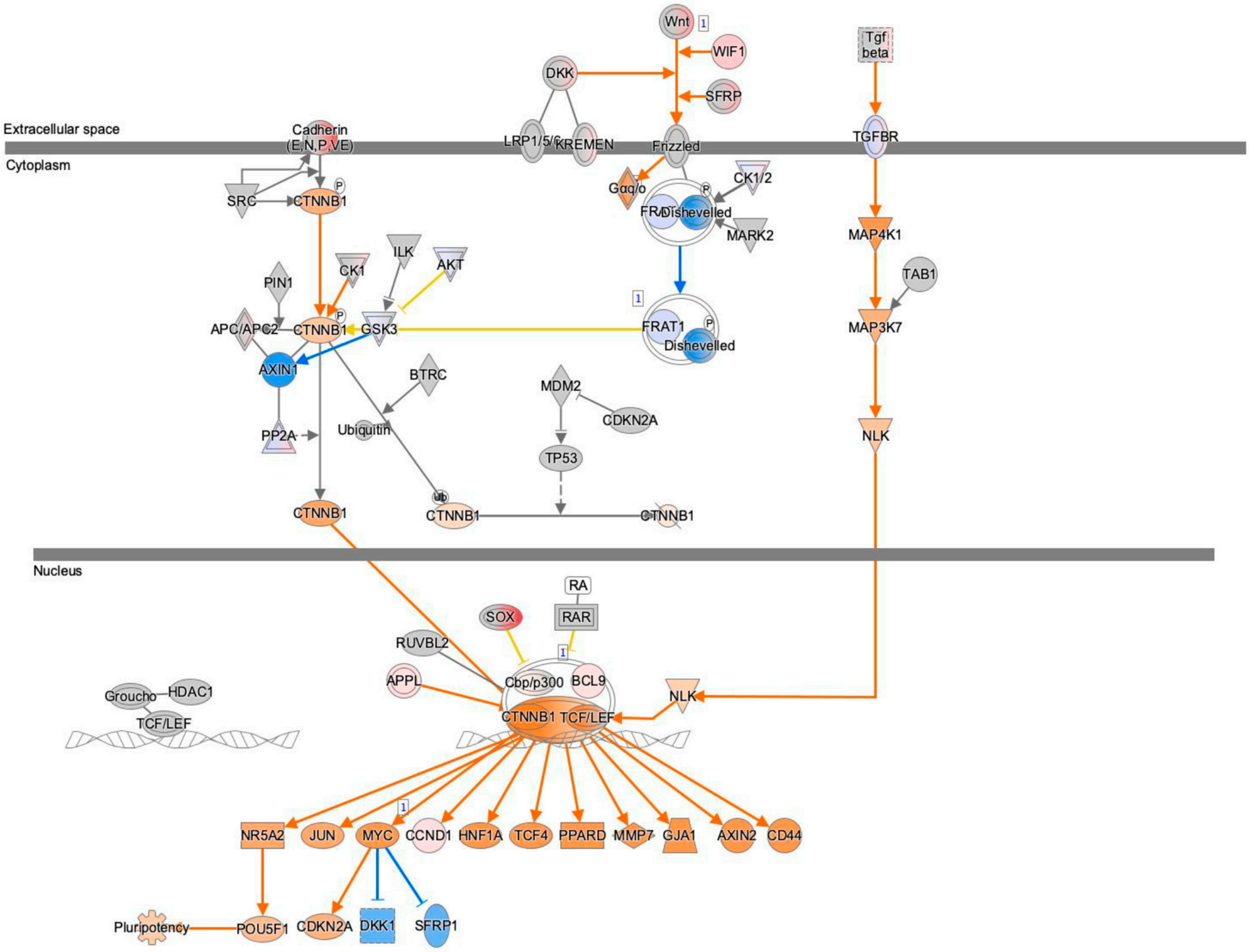

Supplementary Figure S9. Wnt/β-catenin pathway was predicted to be activated by RSV

The figure displays Wnt/β-catenin pathway in which the genes in the data set were overlapped to visualise the effect of RSV intervention on genes implicated in the pathway. The nodes in blue and red/orange represent down-and up-regulated genes respectively. Genes in blue and red/orange nodes are in the data set, and they are significantly dysregulated ($p<0.05$) while genes in grey nodes are also in the data set, but they are not significantly changed.

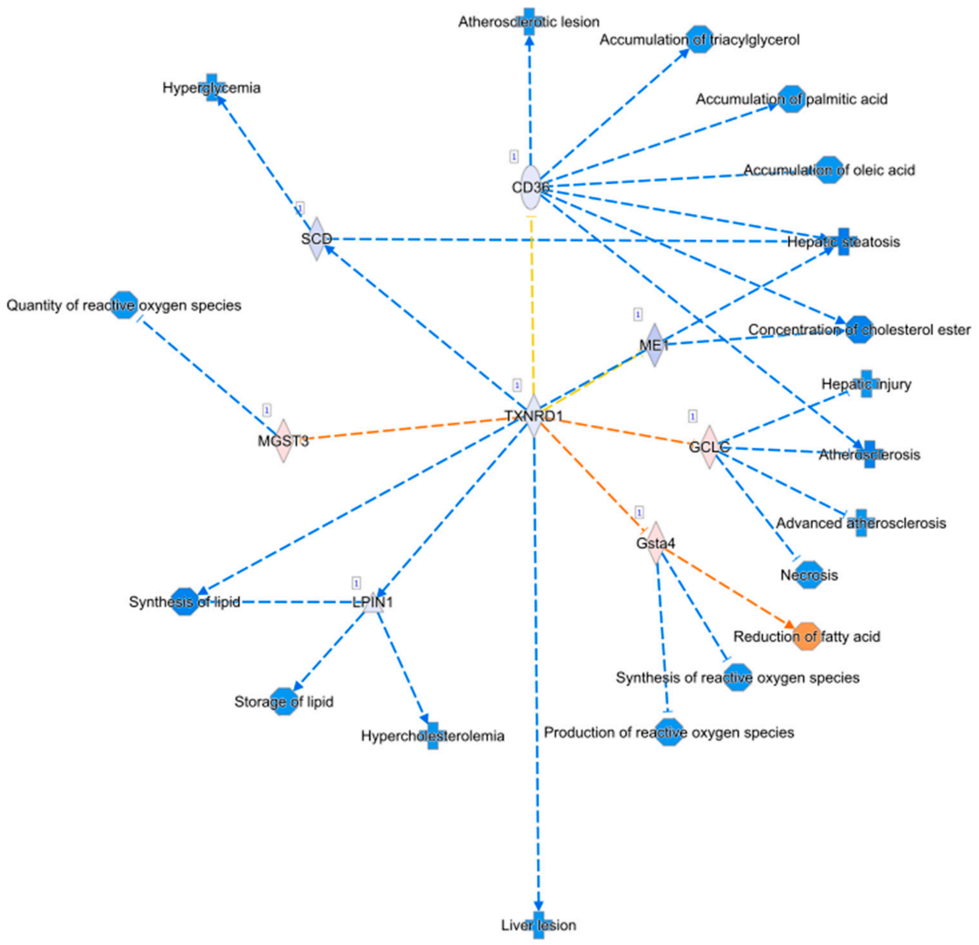

Supplementary Figure 10. Schematic representation of the relationships between thioredoxin reductase-1 upstream regulator and affected genes.

The figure displays the relationship between thioredoxin reductase-1 (Txnrd1) and its target genes that are associated with various atherosclerosis and metabolic disorders. The blue lines and nodes indicate predicted down-regulation effect while the orange and yellow lines and nodes indicate predicted up-regulation effect. Dotted lines indicate an indirect relationship (there is intermediate molecule or step).
